# Electrophysiological correlates of interference control in the modified emotional Stroop task with emotional stimuli differing in valence, arousal, and subjective significance

**DOI:** 10.1371/journal.pone.0258177

**Published:** 2021-10-14

**Authors:** Kamil K. Imbir, Maciej Pastwa, Joanna Duda-Goławska, Adam Sobieszek, Marta Jankowska, Aleksandra Modzelewska, Adrianna Wielgopolan, Jarosław Żygierewicz

**Affiliations:** 1 Faculty of Psychology, University of Warsaw, Warsaw, Poland; 2 Faculty of Physics, Biomedical Physics Division, Institute of Experimental Physics, University of Warsaw, Warsaw, Poland; Universidad Complutense Madrid, SPAIN

## Abstract

The role of emotional factors in maintaining cognitive control is one of the most intriguing issues in understanding emotion-cognition interactions. In the current experiment, we assessed the role of emotional factors (valence, arousal, and subjective significance) in perceptual and conceptual inhibition processes. We operationalised both processes with the classical cognitive paradigms, i.e., the flanker task and the emotional Stroop task merged into a single experimental procedure. The procedure was based on the presentation of emotional words displayed in four different font colours flanked by the same emotional word printed with the same or different font colour. We expected to find distinct effects of both types of interference: earlier for perceptual and later for emotional interference. We also predicted an increased arousal level to disturb inhibitory control effectiveness, while increasing the subjective significance level should improve this process. As we used orthogonal manipulations of emotional factors, our study allowed us for the first time to assess interactions within emotional factors and between types of interference. We found on the behavioural level the main effects of flanker congruency as well as effects of emotionality. On the electrophysiological level, we found effects for EPN, P2, and N450 components of ERPs. The exploratory analysis revealed that effects due to perceptual interference appeared earlier than the effects of emotional interference, but they lasted for an extended period of processing, causing perceptual and emotional interference to partially overlap. Finally, in terms of emotional interference, we showed the effect of subjective significance: the reduction of interference cost in N450 for highly subjective significant stimuli. This study is the first one allowing for the investigation of two different types of interference in a single experiment, and provides insight into the role of emotion in cognitive control.

## 1. Introduction

Each day, we are flooded by an ‘informational tsunami‘ and forced to process plenty of stimuli. However, our cognitive capabilities have their limits [1]. To achieve our goals and focus on important stimuli, we need a special mechanism, i.e., cognitive control. It is a mental ability, combined from distinct and autonomous, but somehow correlated subcomponents [2,3]. Cognitive control helps us to concentrate on the relevant incentives while ignoring the non-significant ones. A recent review of brain imaging data suggests that the essential factors of cognitive control are shifting, which is the ability to flexibly change between task-sets or goals; updating, monitoring and changes to working memory; and inhibition, which helps to suppress the automatic or prepotent response [2]. Among the critical inhibitory processes, interference control has to be discussed. Generally speaking, this mechanism is responsible for suppressing the stimulus that entails a competitive response and suppressing the distraction that can slow down current working memory operations.

In the current study, we distinguished between two types of interference control measured in different paradigms [4]. Interference control may be perceptual, i.e., based on an object’s perceived characteristics (e.g., the physical similarity of letter shapes). This type of interference is present, for example, in the flanker task [5]. However, interference control may also be associated with a stimulus’s meaning (e.g., a word eliciting high arousal). In this way, it is inevitably combined with emotional functioning. This type of interference control can be measured, for example, in the emotional Stroop task (EST) [6]. In the current study, we wanted to examine whether emotional factors such as valence, arousal (the physical form of activation associated with emotions), and subjective significance (the reflective form of activation related to emotions) influence interference control at perceptual or conceptual levels [7]. Emotional interference is in this case indistinguishable from conceptual interference, as the words used as experimental stimuli were loaded with a particular emotional charge. Event Related Potentials (ERP) measures are suitable for this aim because they allow careful investigation of changes associated with specific emotional factors.

### 1.1. Emotional factors influencing interference control

A stimulus’s emotional load can be described along several dimensions, among which valence and arousal seem to be two critical and separate components [8]. Valence refers to the experience of pleasantness vs. unpleasantness of the stimuli, resulting in an approach or avoidance reaction, whereas arousal indicates the level of bodily activation induced by stimuli exposition [9–11] and its biological aspects appraisal [12]. Interestingly, both mentioned components can be treated as orthogonal elements constituting an emotional experience [8,13]. Therefore, manipulation of one factor while controlling the other is possible and can be used in cognitive control tasks (e.g. [14,15]). Valence describes the evaluation of the stimuli as negative, positive, or neutral. In other words, the unpleasantness vs. pleasantness of the emotional reaction. Research has shown that emotionally loaded stimuli are processed faster than neutral ones as they engage more attentional resources [16]. Additionally, human perception is biased to detect possibly dangerous stimuli. Therefore, negatively loaded stimuli capture more attention than positive ones as this is crucial for survival [17,18]. Arousal manifests a level of energy induced by an emotional reaction, and it is related to the automatic, experiential system based on Epstein’s typology [19]. Arousal tends to be a more biologically driven, survival-oriented reaction. Therefore, highly arousing stimuli provoke malfunctioning of complex cognitive processing, such as, for example, cognitive control [20].

However, one should consider the relationship between valence and arousal more carefully. Data suggest that the reported emotional load of words on arousal and valence scales remain in relation best described by a quadratic function [20,21]. Stimuli characterised as highly positive or highly negative are perceived as more arousing than neutrally valenced ones (e.g. [22,23]). The findings discussed above question the assumption of the orthogonal relationship between the related components. Therefore, it seems crucial to select verbal materials with caution, respecting the association between both factors and adjusting the arousal level to ensure uniform comparisons such as highly arousing negative stimuli compared to highly arousing positive stimuli [24].

Another component describing the emotional experience is the subjective significance, comparable to arousal as it is a type of activation resulting from reflective processes [20,25]. Subjective significance refers to the importance assigned to the stimulus from the perspective of the individual’s goals and needs. It might tend to evoke more demanding and energy-consuming systematic cognitive processing [25]. Subjective significance is a similar phenomenon to will-power [26] or the salience concept [27]. Similarly, as arousal activates the experiential mind system, subjective significance is associated with the second, rational (or reflective) system [19,25].

The influence of valence and arousal in cognitive control tasks (like the flanker task or the EST) has shown that emotionally loaded words tend to provoke longer response times compared to neutral ones [6,28]. Firstly, in the classical flanker task, where a positive mood was elicited, more significant interference and slower reaction times were observed than for negative or neutral mood conditions [29]. Additionally, some research used an emotionally modified flanker task where participants were asked to distinguish between the flanker and target stimulus with different arousal levels. It was found that, in a congruent condition (flanker and target stimuli were expressing the same affect), the reaction time was faster compared to incongruent trials (see for example [30–32]). Past results suggest that valence provokes interference in this type of task. The influence of valence and arousal on cognitive control were observed in Van Steenbergen, Band, and Hommel’s experiment [33]. Different types of moods were activated by listening to music and evoking positive memories. Moreover, valence significantly affects cognitive control in tasks, such as the EST, flanker task, and Simon task. In the case of arousal, it was observed that highly arousing verbal stimuli result in emotional interference separately from the valence effect [13]. Additionally, research has shown that the arousal induced by physical activity supports cognitive processing in congruent trials but undermines it in incongruent trials [34]. The influence of arousal on cognitive control was also demonstrated in a study using the recall of emotionally loaded words [35,36]. At the same time, data illustrating the effect of subjective significance on cognitive control is still missing [29].

### 1.2. Electrophysiological correlates of interference control

#### 1.2.1. Flanker task

The flanker task is designed to verify the cognitive charge required for processing [5]. In this task, stimuli presented to the participant are surrounded by the same (in congruent trials) or other stimuli (in incongruent trials). In some studies, neutral conditions are also used. In this case, the distractors flanking the central stimulus are not related to it [37]. The participant is required to respond to the central stimulus, ignoring the flanking stimuli. It has been shown that, on the behavioral level, task solving efficiency is influenced only by the congruency between the central stimulus and the flanker. Namely, incongruent trials have been found to cause slower responses than congruent trials [29,30,37–41]. This effect is caused by the conflicting responses to the tasks. In incongruent conditions, the flankers prime the actual response, generating conflict observed in the response times.

The conflict between responses can also be observed in neural activity. Some authors suggest that the N200 component observed in the anterior parts of the scalp may be the first indicator of visual conflict in the flanker task [40,42,43]. Others argue that in the early period of 200–350 milliseconds after stimulus onset, another component may indicate the visual processing of the task, namely the P2 (or sometimes P200) component [44]. P2 is a positive potential observed from the frontal to parietal areas of the scalp. The differences between processing congruent and incongruent trials have been frequently reported within this component [42,44–47]. Results of an interesting experiment using the flanker task have shown, that processing the incongruent trials evoked larger amplitudes than processing the congruent ones in the P2 component, which in this study was identified as signal recorded over fronto-central areas in the time-period of 150–250 ms from stimulus onset [48].

The late positive complex (LPC) has also been observed to vary according to the flanker’s congruency. LPC is a positive going wave observed over the parietal parts of the scalp about 400 milliseconds after the onset of the stimulus. This component has been originally tied to the processes related to memory [49,50], but effects within this component in the flanker task have been frequently reported [51,52]. Incongruent trials have been reported to evoke more positive potentials than congruent ones [53]. Taking into consideration the late time of the differences between incongruent and congruent trials in the LPC component and the associations of the component to the memory processes, it could be argued that the effects observed in the flanker task in LPC are caused by the decision-making process, which requires comparing the viewed stimuli with the previously remembered rules of the task [54,55].

Manipulating the flanker task’s affective properties has been frequently reported to have an effect on behavioral characteristics [29,36,38,56,57]. When emotional valence is concerned, a negative affect has been reported to decrease the accuracy in the task [56,58], while positive affect has been reported to slow down reaction times [29,57]. High arousal has been reported to decrease the accuracy of the flanker task [56,59]. However, some studies report that both high and low arousal can cause interference in processing the task [39]. Some studies report that high arousal may increase the speed of solving the task [59–61], while others report that both high and low arousal can slow down the processing of the task [39,62]. The subjective significance is an emotional dimension, which has not yet been extensively explored. However, a study using emotional stimuli significant to the participants showed that such stimuli could increase the interference in the flanker task [63].

Some authors suggest a difference between the influence on the flanker task of task-relevant vs. task-irrelevant emotional stimuli [64]. The most common way of using the task-relevant ones is employing the procedure of the emotional flanker task [30,65]. This procedure uses emotional stimuli contrary to arrows or geometric shapes used in the classic approach to the flanker task [5]. The stimuli used in the emotional flanker task could be pictures [30,65] or emotional words [66–68]. Analogically to the classic flanker task, negative stimuli have been reported to decrease the performance in the task [68].

Emotional factors also influence the components of ERPs during the processing of the flanker task or emotional flanker task. The valence dimension was reported to differentiate the amplitudes within the P200 component, namely positive stimuli evoke more positive potentials than the negative ones [69]. The context of safety, which could be connected to the positive valence of emotions and relatively low arousal, was also reported to evoke more positive P200 amplitudes in the flanker task than in the threat context, which could be interpreted as negative and highly arousing [70]. The stimuli presenting people from the same race as the participant, which could be interpreted as more positive and more significant stimuli, were also reported to evoke more positive amplitudes than the stimuli presenting people of different races [71]. Valence was also reported to influence the early posterior negativity (EPN) component. EPN is a negative-going wave observed in the posterior parts of the scalp at around 200–350 ms after stimulus onset. Namely, the negative stimuli have been reported to evoke more negative potentials than neutral ones [72]. Also, in the LPC component, positively valenced stimuli have been reported to evoke more positive potentials than negative ones [69].

#### 1.2.2. Emotional Stroop task

The EST is a modification of the classical Stroop task [73], which measures interference control [4]. As in the classical Stroop task, the participant’s primary task in EST is to name the font color of the presented word. The difference is the nature of the interference, which in this case is caused by the emotional load of the word [4,74]. The target words in the EST are carefully chosen to differ only in emotional factors (e.g., valence, arousal, or subjective significance) while being matched concerning other relevant properties (e.g., frequency, length, or grammatical class). This allows us to infer that the behavioral slow-down and ERP effects observed reflect the automatic attraction of attention caused by the word’s emotional load [24,75].

EST is a useful procedure in studies involving participants with clinical disorders, such as depression, anxiety and PTSD [76]. This procedure is based on recognising the emotional traits of a word, which causes the interference in processing. The emotional charge causing the interference may be amplified in clinical groups when the meaning of the word is related to the objects evoking anxiety, stress or depressive states [13,77,78]. The results of EST may differ between normal participants and trauma survivors, even if the trauma itself did not cause clinical disorders, which was observed on the difference between adults raised in biological families and orphanages [79]. Some researchers even suggest, that with a particular choice of words EST may be used in predicting the possibility of self-harm and suicide [80].

Past ERP studies investigating the effects of the emotionality of words in EST focused primarily on valence. It is first worth noting that emotional words are processed differently from other, more salient emotional stimuli. While processing emotional scenes and faces modulates very early ERP components, emotional word processing has a much more pronounced effect on later ERP components associated with semantic analysis [81]. As one comprehensive review of EEG and fMRI data [82] reports, most cited studies on emotional word processing show emotional effects (e.g., more negative amplitudes for negative relative to neutral words) starting at the 200–300 ms time-window.

The first effect, reported in studies investigating the effect of valence on ERPs, is the increase in occipitotemporal negativity for both negatively and positively valenced words relative to neutral words called the early posterior negativity (EPN) effect. This effect has been reported in silent word reading [83–85] and lexical decisions [84,85].

Valence effects on EPN have been reported to start as early as 100 ms (e.g., [86]). However, as such early potentials are mainly influenced by orthographic features rather than semantic analysis [87], these findings may only reflect conditioned associations with the visual characteristics of valenced, high-frequency words [88], or represent a spillover effect of the block design, in which past conditions influence early potentials on the next stimulus [89].

The P2 component presents a less regular pattern of valence effects, as it has been reported to be sensitive to negative words only [90], positive words only [91], or both positive and negative words [92,93], generally producing a more positive amplitude relative to neutral words.

The N450 is the first component specific to the EST and is associated with cognitive control. It is observed in a 350–500 ms time window in fronto-central areas, sometimes taking the shape of global negativity [94,95]⁠. It was found to be sensitive to the valence of presented words, enhancing negative amplitude for negative words [94,95], and correlating to a more general increase in amplitude while experiencing emotional interference [96–98]. Both P2 and N450 components tend to reaffirm and resemble the behavioral results in EST, presenting a more sensitive measure of inhibitory control [98,99].

The effects of valence on the LPC are somewhat inconsistent. Studies of silent word reading and lexical decisions report either that the processing of negative words evokes more positive LPC amplitudes than neutral or negative words [7,83,88,93,100,101], or just the opposite pattern of results [84,85,102,103], but as LPC is claimed to be a manifestation of later stages of semantic processing [104,105] associated with attention and conscious recognition of stimulus [106], one regularity seems to find more and more support in the literature: LPC becomes more emotionally modulated as the level of attention to the valence of the word increases [107,108]. Specifically, González-Villar et al. [107] conducted a study using the EST and a task where participants had to judge the emotionality of words and found that the LPC was modulated by valence only in the latter one. This finding was later replicated by Delaney-Busch, Wilkie, and Kuperberg [109] under different task demands. Similarly, our previous EST study [98] found no valence effect on the LPC.

The literature on the effects of arousal on LPC is less robust than that of valence. The study mentioned above by Delaney-Busch et al. [109] found that while valence did not modulate the LPC in a task in which participants judged whether a word denotes an animal, an increase in the amplitude of the LPC was observed for high arousal words, relative to words with a low level of arousal.

As for earlier components, our previous ERP study in the EST paradigm [98] found that the P2 component was modulated by arousal, exhibiting a more positive amplitude for high arousal words when compared to moderate arousal words. Similar previous results include those of Thomas et al. [99], who found that threat-related words elicit more positive amplitude P2 responses than neutral words.

Subjective significance, being the factor that only recently started to be examined experimentally, is most lacking in systematic empirical studies. Research has already indicated its influence on ERP components. For example, Herbert et al. [110] showed that emotional stimuli self-referentiality correlated with greater LPC amplitudes. Recently, we have demonstrated [111] that subjective significance influences even earlier components, such as FN400. In a lexical decision task experiment, amplitudes for highly significant words were more positive than for words of low subjective significance while controlling for arousal and valence. Because such a task employs deeper processing of words than the EST and, as we discussed, the strength of ERP effects tends to resemble the depth of processing and attention to emotional factors, it remains an empirical question whether subjective significance modulates components related to inhibitory control in the EST. Preliminary behavioral results showed that subjective significance shaped reaction times [25]. Even more promisingly, in an EST experiment with a much shorter word list and not examining valence effects [98], we found that subjective significance modulated the N450 and even the P2 components. The P2 amplitude was more positive for moderately significant words relative to highly significant words. The N450 amplitude was more positive for highly significant words as compared to minimally significant words.

#### 1.2.3. Merging the flanker task with the emotional Stroop task

Many procedures have been proposed that merge the emotional flanker task with the EST [33,112]. In different variations of this task, the emotional word, written in a specific colour, is either placed next to the flanker word [113] or surrounded by flanker words [113–117]. Some researchers also use separate screens, including and excluding flankers with a short break between them [117]. The words used as flankers could be the names of the colours [33,113] or the same words as the target written in either the same colour (congruent condition) or a different colour (incongruent condition) [113,115–117]. No matter the exact structure of the displayed trials, the participant’s task is always to name the colour of the target word, as in the EST.

Combining the two tasks has been confirmed as a procedure revealing the influence of emotional charge on cognitive control. It has been reported that emotions speed up the processing of incongruent trials, both negative [114,118] and positive ones [116,119]; some suggest that negative emotions may be more impactful [33,117]. These results suggest that both valence and arousal may speed up processing in this kind of task. As for the ERP results, the N200 component, usually involving differences in the flanker task, also turned out to be stimulated by emotions in the combination of flanker and Stroop tasks [43,118,119]; however, some studies also observed differences in the N450 component [113] typical to the EST. We hypothesise that, in this combination of two tasks, we could expect the effects related to processing both the flanker task and EST, as this task includes incongruence on the purely visual level and emotional stimulation on the level of the meaning of the word.

### 1.3. Aim and hypothesis

In the current experiment, we aimed to investigate the electrophysiological correlates of two different types of cognitive control that can be measured in the combined flanker and Stroop tasks: perceptual and conceptual (associated with meaning) levels of interference control [7]. In the flanker test, the interference is based on the perceptual features of the stimuli. In contrast, in the EST, the interference is based on stimuli meaning and emotional connotations. A high emotional load is thought to capture attention for stimulus processing, and thus it is harder to get the correct answer for an untrained task (naming the colour of the text). On the behavioural level, we expected both flanker incongruence and the affective features of words to impose interference costs and thus lengthen reaction times. Considering emotional factors, we predicted that increasing the arousal level would cause increasing difficulty in maintaining interference control (thus longer reaction latencies would be observed). We also predicted that increasing levels of subjective significance, a factor introduced as an activation mechanism for reflective and effortful processing, would increase the effectiveness of interference control, thus reducing reaction times.

The measurement of ERP correlates of cognitive processing gives us a unique chance to investigate, precisely in time, the course of changes evoked by the different types of cognitive interference (perceptual and conceptual). Since the analysed issue is relatively new in the literature, we decided to analyse the data using two different approaches: exploratory and classical component-based. Considering the exploratory approach, in general, we expected the effect of flanker congruency (perceptual features inhibition) earlier than the effects of emotional factors (conceptual meaning features interference). The amplitude in conditions characterising greater interference (e.g., incongruent flanking stimuli) was expected to be augmented (i.e., more negative or more positive, depending on the amplitude general tendency) in comparison to conditions characterising lower interference (e.g., congruent).

Considering the classical component-based approach, we expected to find the flanker congruency effect in EPN, i.e., more negative amplitudes for incongruent stimuli in comparison to congruent stimuli. We also expected to find arousal and subjective significance effects in the P2 and N450 components. Arousal was expected to impair inhibition control effectiveness and amplitudes were expected to be augmented (more positive in P2 and more negative in N450) for high arousal words conditions than low arousal words. Subjective significance was expected to improve the effectiveness of inhibition control. Thus, amplitudes were expected to be less augmented (less positive in P2 and less negative in N450) for the conditions with high subjectively significant words than low subjectively significant words. Finally, we expected to find a valence effect in LPC component amplitude, i.e., differentiation between negative and positive valence categories of words, since LPC indexes the meaning of word processing and discrimination of different categories [82].

We also expected interactions between the manipulated variables. However, because the current experiment was the first in the field allowing us to investigate this, we had no specific expectations, and we have treated this part of our work as pure exploration.

## 2. Materials and methods

### 2.1 Participants

The participants were recruited from various faculties of Warsaw universities. They had to meet the following criteria to be included in the experimental group, i.e., they had to be right-handed, native Polish speakers, without chronic clinical issues that may affect EEG recording directly or through medication (e.g. neurological and mental disorders). The participants had their vision intact or corrected to normal by glasses. They received a small compensation for taking part in the experiment. The entire experimental group consisted of 36 participants (18 men and 18 women), from 19 to 28 years old (*M* = 21.78; *SD* = 2.40). After collecting the data, some participants were excluded from EEG analyses if, due to excessive artefacts or extremely short or long response time, they had more than 50% of trials rejected. Effectively, there were 34 participants included in the further analysis, 18 men and 16 women, aged 19–28 years (*M* = 21.88; *SD* = 2.42).

Based on our previous research using similar procedures with the same emotional factors (Imbir et al., 2021), as well as other recent research (Citron et al., 2013; González-Villar et al., 2014), we estimated, that the expected effect sizes could be from *η*^*2*^ *=* .1 to *η*^*2*^ *=* .21 for simple effects and from *η*^*2*^ *=* .1 to *η*^*2*^ *=* .28 for interaction effects. We conducted a-priori power analysis using G-Power software (Faul et al., 2009), estimating that the number of participants needed to achieve a high statistical power of the study (at least .8) for the interaction of two factors would be at least 18 participants. The design of the study, with a large number of repeated measures for each emotional factor, ensures the high statistical power, while a larger number of participants (initially double the estimated number) allows us to observe an interaction of more than two factors–such interactions, however, are burdened with smaller statistical power.

We did not collect any personal data that would allow for the identification of the participants. The participants provided written informed consent to participate in the experiment, which was documented and stored in the research diary. The bioethical committee of the Faculty of Psychology at the University of Warsaw approved the design, experimental conditions, and procedure. All of the procedures involving human participants were done following the ethical standards of the institutional and national research committee and with the 1964 Helsinki Declaration and its later amendments or comparable ethical standards.

### 2.2 Design

We investigated the behavioural and electrophysiological measures related to interference control while performing a flanker task combined with EST. We manipulated the factors of valence (3 levels), arousal (3 levels), and subjective significance (3 levels) while controlling the following properties of words: concreteness, frequency of appearance in language, and length. There were also two levels related to the flanker task, i.e., the congruent or incongruent colours of stimulus vs. flankers.

### 2.3 Linguistic materials

#### 2.3.1 Word selection

As the word stimuli, we used 405 nouns acquired from the Affective Norms for Polish Words Reload database [120]. In the process of validating this database, each word had been assessed on a self-assessment manikin scale [121] by 50 participants (25 women and 25 men) on eight different dimensions (Valence, Arousal, Dominance, Origin, Significance, Concreteness, Imageability, and Age of Acquisition). Mean values for every dimension were calculated for each of these words.

Words were divided into 27 groups (15 words each) by their valence (negative, neutral, and positive), arousal (low, moderate, and high), and subjective significance (low, moderate, and high). We also controlled for two other factors, namely the length of the words (the number of letters) and the frequency of usage in the Polish language, transformed into natural logarithms [122].

For the dimension of valence, mean ratings of the experimental stimuli were *M* = 3.98, *SD* = 0.54 for negative, *M* = 5.12, *SD* = 0.22 for neutral and *M* = 6.15, *SD* = 0.46 for positive words. As for the arousal, stimuli of low arousal had mean ratings *M* = 3.34, *SD* = 0.26, moderate arousal words were *M* = 3.98, *SD* = 0.15 and words of high arousal were *M* = 4.75, *SD* = 0.41. In subjective significance, words of low significance were *M* = 3.0, *SD* = 0.28, these of moderate significance *M* = 3.62, *SD* = 0.14, and stimuli of high subjective significance were *M* = 4.36, *SD* = 0.39. The list of words used in the experiment may be found in S1 Appendix.

We conducted ANOVA in a 3 (levels of valence) x 3 (levels of arousal) x 3 (levels of subjective significance) model for all dimensions (including also the two controlled ones), verifying the accuracy of experimental stimuli selection. To justify stimuli selection, we should obtain significant effects of valence levels on valence ratings only (treated as the dependent variable), effects of arousal on arousal ratings, and effects of significance on significance ratings. There should be no more significant effects, therefore indicating that groups were different on the experimental dimensions only.

As expected, we found significant differences between groups of valence divided by valence rating: *F*(2, 396) = 878.65, *p* < 0.001, *η*^*2*^ = 0.82. There were no significant effects for valence divided by other experimental dimensions, namely for the arousal ratings: *F*(2, 396) = 1.60, *p* = 0.2, *η*^*2*^ = 0.008 and for subjective significance ratings: *F*(2, 402) = 0.49, *p* = 0.61, *η*^*2*^ < 0.01. There were also no significant differences between groups of valence on controlled dimensions: *F*(2, 402) = 0.65, *p* = 0.52, *η*^*2*^ < 0.01 for the frequency of usage in the Polish language: *F*(2, 402) = 1.09, *p* = 0.34, *η*^*2*^ < 0.01 for the number of letters.

On the dimension of arousal, we found only a significant effect for groups of arousal divided by arousal ratings: *F*(2, 396) = 775.90, *p* < 0.001, *η*^*2*^ = 0.80. We did not find any statistically significant effects for arousal on the scale of valence: *F*(2, 396) = 0.69, *p* = 0.5, *η*^*2*^ = 0.003, or subjective significance: *F*(2, 402) = 0.025, *p* = .98, *η*^*2*^ < 0.01. Again, there were no differences between groups of arousal on the frequency of usage in the Polish language: *F*(2, 402) = 0.68, *p* = 0.5, *η*^*2*^ < 0.01 or for the number of letters: *F*(2, 402) = 0.06, *p* = 0.98, *η*^*2*^ < 0.01.

For subjective significance, there was only an effect for the groups of significance divided by significance ratings: *F*(2, 396) = 747.32, *p* < 0.001, *η*^*2*^ < 0.798. There were no significant effects for valence ratings: *F*(2, 396) = 2.27, *p* = 0.10, *η*^*2*^ = 0.01, or arousal ratings: *F*(2, 396) = 0.12, *p* = 0.89, *η*^*2*^ = 0.001. Also, for this dimension, there were no differences between groups on the frequency of usage in the Polish language *F*(2, 402) = 0.92, *p* = 0.72, *η*^*2*^ < 0.01 or the number of letters: *F*(2, 402) = 1.18, *p* = 0.15, *η*^*2*^ < 0.01.

### 2.4 Procedure

The participants sat in a comfortable chair. The words were displayed on a 17.3-inch diagonal LCD, at a distance of approximately 1 m from the participant’s eyes. The font was Helvetica, a size of 10 percent of the screen height. Participants were encouraged to respond as quickly and as accurately as possible.

The task was to assess the font colour of the middle word by pressing tagged keys on the keyboard. Above and below the target stimuli, there was the same word but printed in either a congruent or incongruent font colour. The content and latency of the response were recorded. A single experiment consisted of two runs of comprised 810 trials, i.e., 15 words in each of 27 categories (3 arousal levels x 3 valence levels x 3 subjective significance levels, repeated in two conditions (congruent and incongruent). The categories were presented in blocks. The order of the categories in each run was randomised. The order of words within a block and order of congruent/incongruent conditions was also randomised but subjected to the condition that the same word could not be presented in successive trials. A trial proceeded as follows:

1. Fixation cross displayed for a randomly varied interval between 400–500 ms.
2. The stimulus presented until the participant responds, but not shorter than for 300 ms.
3. The blank screen displayed for a randomly varied interval between 1000–1100 ms.

**Fig 1:**
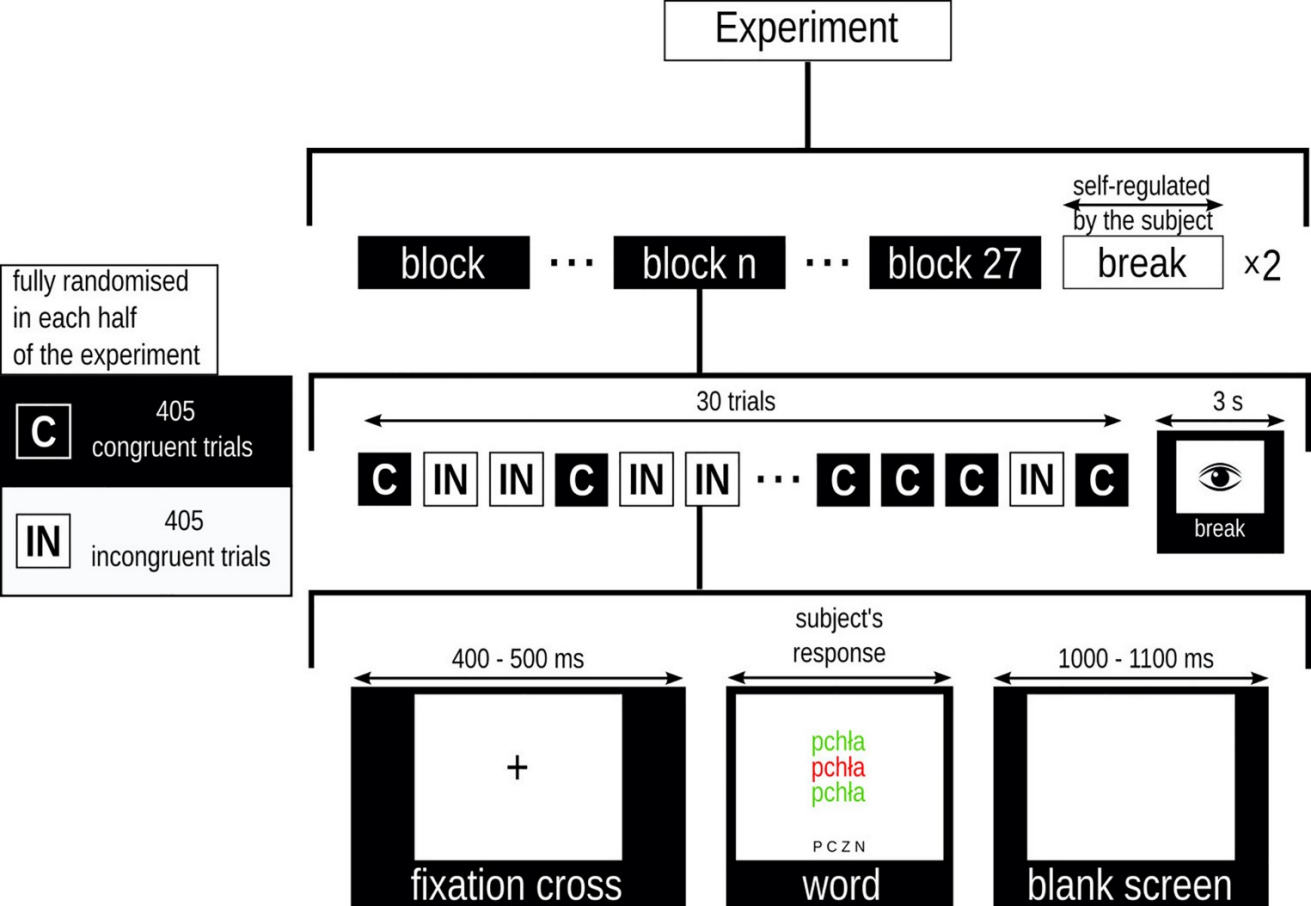
Diagram of the experimental protocol. The task was to assess the font colour of the middle word ignoring the colour of the word above and below the target. In the diagram, an incongruent example is shown.

The experimental protocol provided three-second breaks for normal blinking every 30 trials. A break self-regulated by the participant separated the runs of the experiment. The procedure is outlined in Fig 1.

### 2.5 EEG recording

#### 2.5.1 Apparatus

The stimuli were displayed on a standard personal computer monitor. The stimuli were synchronised to EEG recording by a circuit that recorded changes in the brightness of a small rectangle on display, covered from the participant’s view. Its brightness changed synchronously with the content of the screen. We recorded EEG signals from 19 electrode sites: Fz, Cz, Pz, Fp1/2, F7/8, F3/4, T7/8, C3/4, P7/P8, P3/4, O1/2 referenced to linked earlobes. The ground electrode was placed at the AFz position. All impedances were kept at a similar value below 5 kOhm. The signal was acquired using a Porti7 (TMSI) amplifier, sampled at 1024 Hz.

#### 2.5.2 Offline EEG signal processing

We conducted offline signal processing utilising Matlab® with the EEGLAB toolbox [123] and custom-made scripts. Offline, the signal was zero-phase filtered. We used the second-order Butterworth filters with 12 dB/octave roll-off; the high-pass filter cut-off was 0.1 Hz, the low-pass cut-off was 30 Hz. Additionally, we used the notch filter for the 49.5–50.5 Hz band, also implemented as the second-order Butterworth filter.

We extracted intervals ranging from -200 to 800 ms, with 0 being the onset of the stimulus. The signals were baseline corrected to the interval -200 to 0 ms. We removed from further analysis trials in which the participant did not correctly identify the colour of the presented word.

Additionally, we removed trials with response time shorter than the (Q1—W) or longer than the (Q3 + W) of the response time individually for each participant, where Q1 is the 25th percentile, Q3 is the 75th percentile, W = 1.5*(Q3—Q1). These operations were performed on data transformed by a natural logarithm. Effectively, the response time for the analysed data across all participants is within 295–5700 ms. The mean number of trials per condition was *M* = 28.59, *SEM* = 0.03.

We prepared the data in the following way. Bad channels were identified as those with normalised kurtosis greater than 5. They were removed and interpolated. Because the stimulus consisted of three lines of text, there were many saccades, which could influence the analysis. Therefore, the signals were decomposed into independent components using the runica algorithm. Components related to blinks and saccades were identified and removed using the MARA procedure [124]. The remaining components were used to reconstruct the clean signal at the electrodes.

### 2.6 Statistical procedures

The distribution of response accuracy was not Gaussian; therefore, the significance of effects concerning this variable was assessed using the Kruskal-Wallis test.

The effects concerning other variables, with approximately normal distributions, were assessed using ANOVA with repeated measures in a hierarchical procedure. We investigated behavioural effects (logarithm of reaction time) and the classical EEG component amplitude effects. The significant main effects were analysed with post-hoc paired t-tests with Holm’s correction for repeated comparisons [125]. The significant two-way interactions were similarly investigated using post-hoc paired t-tests with Holm’s correction (we report the corrected p-values). In the case of significant three-way interactions, they were further analysed by a series of two-way ANOVAs with the levels of a selected variable set iteratively to subsequent levels. The selected variables were permuted. The significance of the effects repeatedly appearing in the series was assessed, taking into account the Bonferroni correction for the number of multiple comparisons, but note that for these analyses, we report the uncorrected p-values. The significant two-way interactions were further investigated using post-hoc t-tests with Holm’s correction. In case an effect could be obtained by different paths in the hierarchical analysis, we report the most conservative result.

We also performed an exploratory analysis of the EEG effects. In this case, there were additionally two factors that we had to consider: time-window and region of interest (ROI). On the first level of the procedure, we performed a four-way ANOVA with repeated measures, one for each time window. The significance of the effects repeatedly appearing in the series was corrected for multiple comparisons by the Bonferroni correction.

The mean ERP amplitude within a given time window was the dependent variable, and the independent variables were valence, arousal, significance, and ROI. Similarly, we investigated the interaction effects occurring between the factors at subsequent steps through the analysis of variance, which took as independent variables the interacting factors from a previous step, as for behavioural and classical component-based ERP analysis. We continued the investigation to a level at which one could understand the interactions in terms of differences in effects of simple factors, or by the interaction of two factors, under specific conditions determined by the particular levels of the other factors. We performed the post-hoc analysis using pairwise t-tests. We handled the problem of multiple comparisons by utilising the Holm procedure. We checked the sphericity with Mauchly’s test and applied the Greenhouse-Geisser correction where necessary. The analyses were implemented in the R statistical package [126].

## 3. Results

### 3.1 Behavioural

#### 3.1.1. Response accuracy

We investigated the accuracy of performing the task (*M* = 97.43%, *SEM* = 0.27%). We performed a series of Kruskal-Wallis rank sum tests for each of the design factors. We did not obtain any significant effects, namely the statistics are for valence (*χ*^*2*^(2) = 2.13 *p* > .05), for arousal (*χ*^*2*^(2) = 0.16, *p* > .05), for subjective significance (χ^2^(2) = 0.28, *p* > .05), and for flanker congruency (*χ*^*2*^(2) = 0.56, *p* > .05).

#### 3.1.2 Reaction time

**Fig 2:**
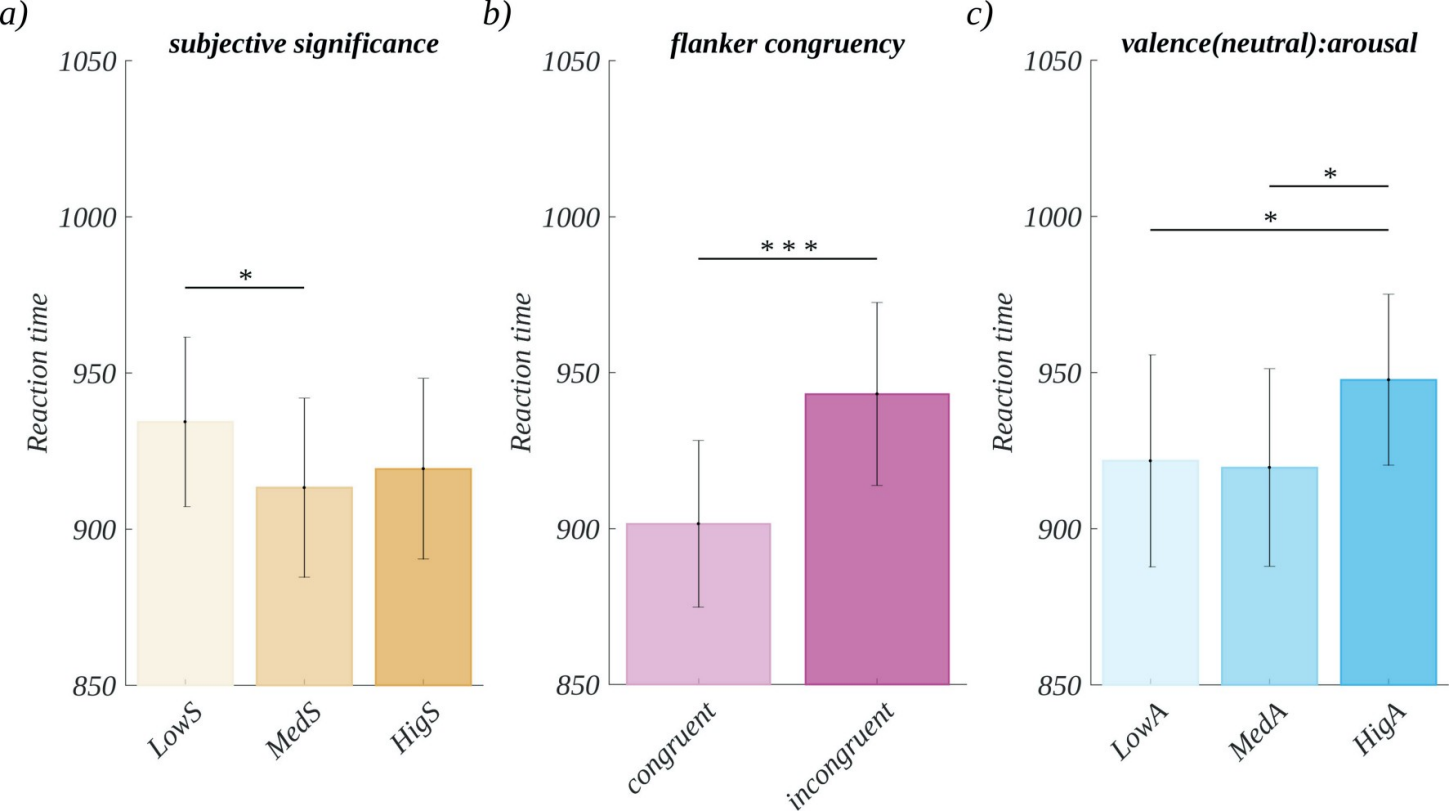
Significant effects found in the reaction times. Main effects of a) subjective significance; b) flanker congruency; c) effect of arousal for neutral words.

We performed ANOVA with repeated measures on the natural logarithms of reaction times. We obtained a significant main effect of subjective significance (*F*(2, 66) = 3.38, *p* = .040; *η*^*2*^ = 0.09). The post-hoc tests showed that, for moderately significant stimuli, the latency (*M* = 913.24 ms, *SEM* = 28.72 ms) was significantly shorter than for low significant words (*M* = 934.33 ms, *SEM* = 27.14 ms; *t*(33) = -2.56, *p* = .046, *d* = -0.89) (Fig 2A). Furthermore, the main effect of flanker congruency also was significant (*F*(1, 33) = 85.56, *p* < .001; *η*^*2*^ = 0.72). Here, the latency for incongruent (*M* = 943.10 ms, *SEM* = 29.32 ms) colours was significantly longer than in the case of congruent colours (*M* = 901.47 ms, *SEM* = 26.73 ms; *t*(33) = 9.25, *p* < .001, *d* = 3.22) (Fig 2B). The main effects of neither valence nor of arousal were found significant.

Moreover, we obtained a significant effect of interaction between valence and arousal (*F*(4, 132) = 2.64, *p* = .037; *η*^*2*^ = 0.07). Further analysis of variance within each level of valence revealed that for neutral stimuli there was a main effect of arousal levels (*F*(2, 66) = 7.21, p = .001; *η*^*2*^ = 0.18). The post-hoc tests showed that latency for high arousal words (*M* = 947.64 ms, *SEM* = 27.38) was longer than for both low arousal (*M* = 921.68 ms, *SEM* = 33.95 ms; *t*(33) = -3.07, *p* = .013, *d* = -1.07), and for moderate arousal (*M* = 919.56, *SEM* = 31.61; *t*(33) = -3.06, *p* = .013, *d* = -1.06) stimuli.

### 3.2 EEG

#### 3.2.1 Exploratory approach

**Fig 3:**
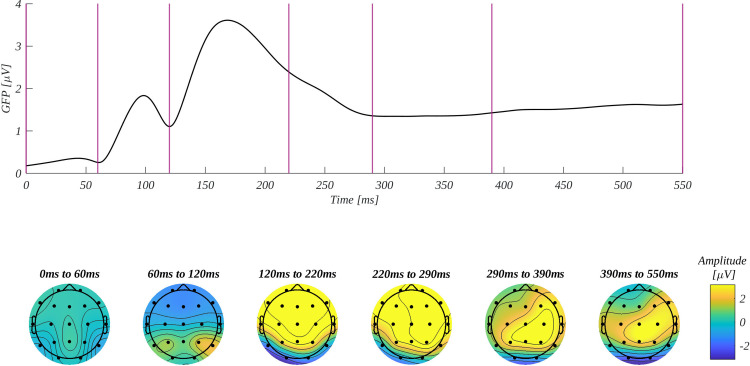
The global field power (upper trace) and topographical distribution of mean amplitude in the selected time-windows (lower part). The vertical lines mark the boundaries of the successive time windows.

**Fig 4:**
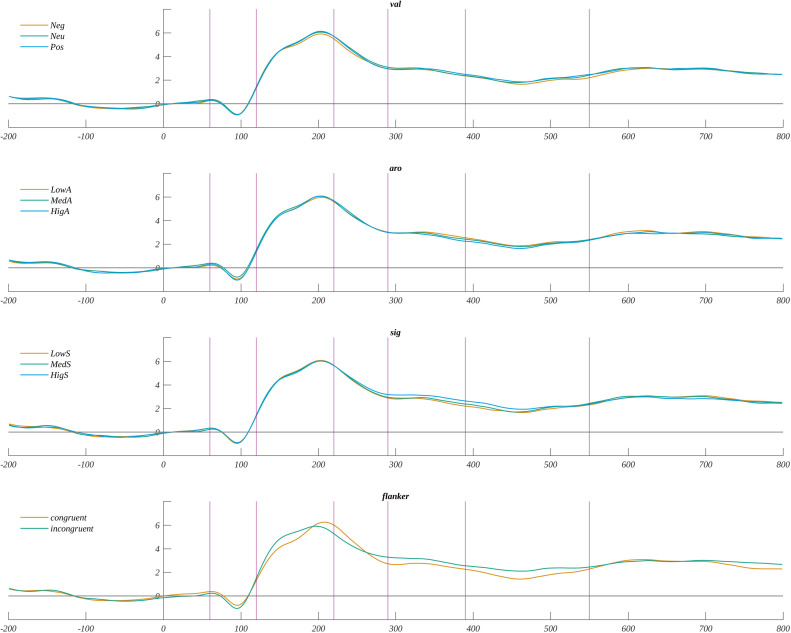
The grand average across all participants and ROIs for each factor and each level. The vertical lines mark the boundaries of the selected time windows. Horizontal axis: time in ms; vertical axis: amplitude in μV.

**Fig 5:**
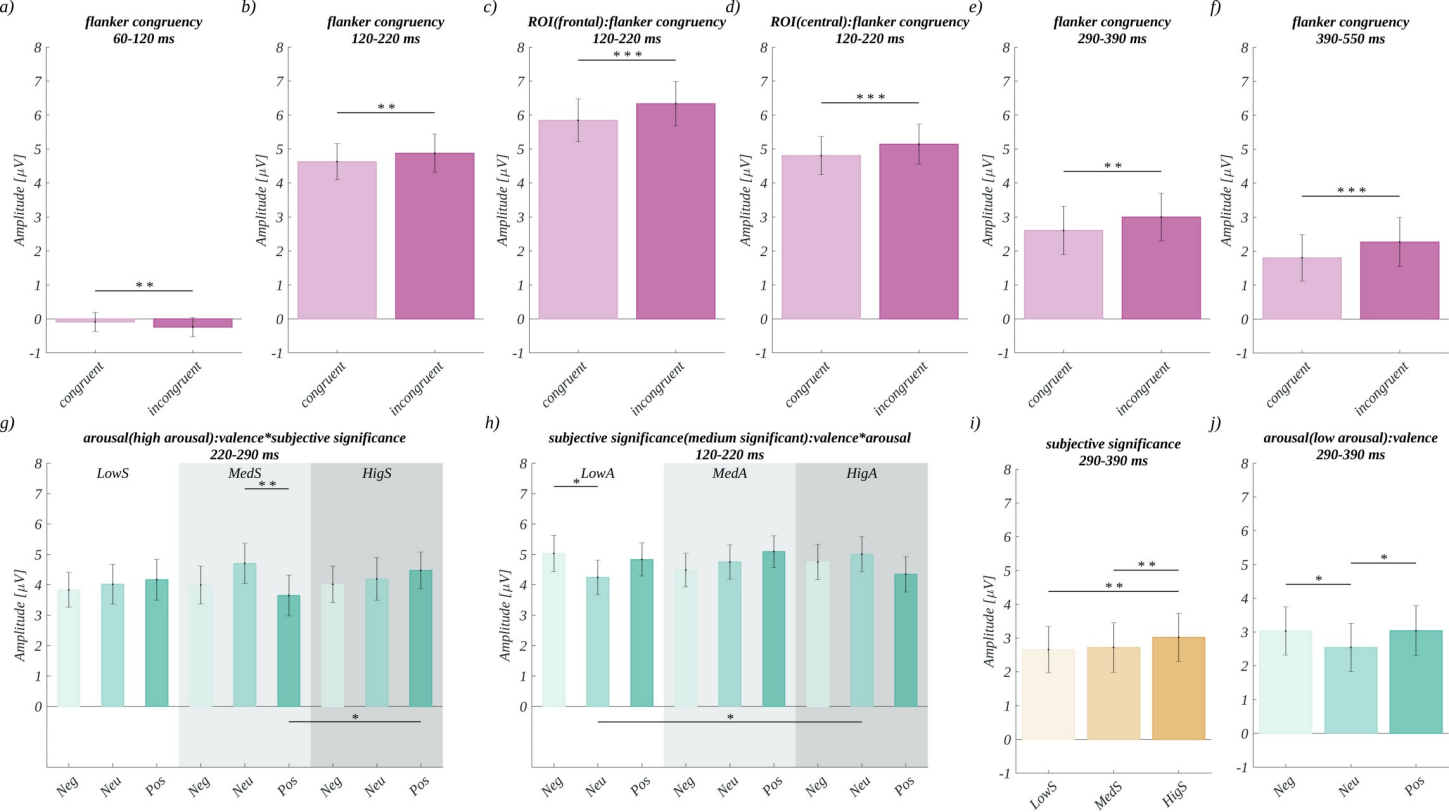
Illustration of significant effects found in the exploratory analysis of ERPs. Main effects of flanker congruency in successive time windows: a) 60–120 ms, b) 120–220 ms. Effect of interaction between flanker congruency and ROIs: c) frontal, and d) central, in 120–220 ms time window. Main effects of flanker congruency in successive time windows: e) 290–390 ms, f) 390–550 ms. g) Interaction between valence and significance for high arousal words in the time window 220–290 ms. h) Interaction between arousal and valence for words of moderately subjective significance in the time window 120-220ms. i) The main effect of significance in time window 290–390 ms. j) Effect of valence for low arousal stimuli in the time window 290–390 ms.

For the exploratory analysis of ERP amplitude, we selected three regions of interest (ROI): frontal (F: F3, Fz, F4), central (C: C3, Cz, C4), and parietal (P: P3, Pz, P4), as they allow the study of possible frontal-posterior asymmetry. Furthermore, we selected five time windows: 60–120 ms, 120–220 ms, 220–290 ms, 290–390, and 390–550 ms. The choice of these ranges was based on the global field power (GFP) curve (Fig 3). Successive minima and inflection points of GFP were used to determine the edges of evoked potential components. The topographic plots of amplitude distribution at the bottom of Fig 3 illustrate the microstates corresponding to the components. The overview of the time course of obtained ERPs is given in Fig 4. We conducted a four-way analysis of variance with repeated measures in successive time windows. The summary of the obtained results is shown in Fig 5. All amplitudes are given in μV. Below we present details of the obtained results. Additionally, in S2 Appendix we show the descriptive statistics and ANOVa results including the non-significant main effects.

For the **time range of 60–120 ms**, we only observed the main effect of flanker congruency (*F*(1, 33) = 9.05, *p* = .005; *η*^*2*^ = 0.22). The amplitude for incongruent colours (*M* = -0.24, *SEM* = 0.28) was significantly more negative than in the congruent case (*M* = -0.09, *SEM* = 0.28; *t*(33) = -3.01, *p* = .005, *d* = -1.05) (Fig 5A). We did not find a main effect of the other factors, i.e., valence, arousal, or subjective significance.

In the **time window of 120-220ms**, we also obtained a main effect of flanker congruency (*F*(1, 33) = 9.90, *p* = .003; *η*^*2*^ = 0.23). The amplitude in the incongruent (*M* = 4.87, *SEM* = 0.56) condition was significantly more positive than in the congruent condition (*M* = 4.63, *SEM* = 0.53; *t*(33) = 3.15, *p* = .003, *d* = 1.10). (Fig 5B). The main effects of other factors were not significant, i.e., valence (*F*(2, 66) = 0.60, *p* = 0.55, *η*^*2*^ = 0.02), arousal (*F*(2, 66) = 0.78, *p* = 0.46, *η*^*2*^ = 0.02), and subjective significance (*F*(2, 66) = 0.41, *p* = 0.67, *η*^*2*^ = 0.01).

Furthermore, an interaction between ROI and flanker congruency (*F*(1.20, 39.74) = 36.51, *p* < .001, *η*^*2*^ = 0.53) was found. Mauchly’s test indicated that the assumption of sphericity had been violated for this factor (Xi2 (2) = 0.34, p < .001), so degrees of freedom were corrected using Greenhouse-Geisser estimates of sphericity (epsilon = 0.60). Further analysis within each ROI revealed that the effect of flanker congruency was significant in frontal (*F*(1, 33) = 29.51, *p* < .001; *η*^*2*^ = 0.47) and central (*F*(1, 33) = 16.10, *p* < .001; *η*^*2*^ = 0.33) ROIs. In both cases, the amplitude for the incongruent condition was more positive than for the congruent condition. Namely, the following statistics were observed: in the frontal ROI, incongruent (*M* = 6.33, *SEM* = 0.65) vs. congruent (*M* = 5.84, *SEM* = 0.63; *t*(33) = 5.43, *p* < .001, *d* = 1.89)(Fig 5C); in the central ROI. incongruent (*M* = 5.14, *SEM* = 0.59) vs. congruent (*M* = 4.80, *SEM* = 0.56; *t*(33) = 4.01, *p* < .001, *d* = 1.40) (Fig 5D).

Moreover, a three-way interaction between arousal, valence, and subjective significance (*F*(8, 264) = 3.21, *p* = .002; *η*^*2*^ = 0.09) was found. Further ANOVA tests within each level of subjective significance showed that, for the moderate level, there was an interaction between valence and arousal (*F*(4, 132) = 5.63, *p* < .001; *η*^*2*^ = 0.15). Post-hoc tests revealed that the amplitude in case of neutral, high arousal words (*M* = 5.00, *SEM* = 0.58) was more positive than for neutral and low arousal stimuli (*M* = 4.24, *SEM* = 0.56; *t*(33) = -3.55, *p* = .041, *d* = -1.24). In case of low arousal, negative stimuli (*M* = 5.03, *SEM* = 0.60) the amplitude was more positive than for low arousal, neutral stimuli (*M* = 4.24, *SEM* = 0.56; *t*(33) = -3.86, *p* = .018, *d* = -1.34) (Fig 5G).

In the **time window of 220–290 ms**, we did not find any significant main effects. However, an effect of the three-way interaction between valence, arousal, and subjective significance was found (*F*(8, 264) = 2.972, *p* < .003; *η*^*2*^ = 0.08). Further analysis within each level of arousal showed that, in the case of high arousal words, there was an interaction between valence and subjective significance (*F*(4, 132) = 4.39, *p* = .002; *η*^*2*^ = 0.12). The post-hoc tests revealed that, for moderately significant words, the amplitude was more positive in the neutral (*M* = 4.70, *SEM* = 0.66) vs. the positive condition (*M* = 3.64, *SEM* = 0.68; *t*(33) = -4.30, *p* = .005, *d* = -1.50). Moreover, for positive words, the amplitude was more positive for highly significant (*M* = 4.47, *SEM* = 0.60) than for moderately significant stimuli (*M* = 3.64, *SEM* = 0.68; *t*(33) = -3.53, *p* = .044, *d* = -1.23) (Fig 5H).

In the **time window of 290–390 ms**, we obtained the effect of flanker congruency (*F*(1, 33) = 12.63, *p* = .001; *η*^*2*^ = 0.28). The average amplitude was significantly more positive in the incongruent (*M* = 2.99, *SEM* = 0.70) than in the congruent condition (*M* = 2.60, *SEM* = 0.71; (*F*(1, 33) = 12.63, *p* = .001; *η*^*2*^ = 0.28) (Fig 5E). Furthermore, the main effect of subjective significance (*F*(2, 66) = 8.46, *p* = .001; *η*^*2*^ = 0.20) was found. The amplitude for words of high subjective significance (*M* = 3.02, *SEM* = 0.71) was more positive than both for words of low subjective significance (*M* = 2.66, *SEM* = 0.68; *t*(33) = -4.39, *p* < .001, *d* = -1.53), and for moderately subjective significance (*M* = 2.72, *SEM* = 0.73; *t*(33) = -3.22, *p* = .006, *d* = -1.12) (Fig 5I). The main effects of the other two factors, i.e., valence and arousal, turned out to be insignificant.

Moreover, we observed a significant effect of interaction between arousal and valence (*F*(2.94, 97.16) = 4.04, *p* = .010, *η*^*2*^ = 0.11). Mauchly’s test indicated that the assumption of sphericity had been violated for this factor (Xi2 (4) = 0.53, p = .021)m so degrees of freedom were corrected using Greenhouse-Geisser estimates of sphericity (epsilon = 0.74).

Further analysis within the levels of arousal showed that the effect of valence was significant for low arousal words (*F*(2, 66) = 4.49, *p* = .015; η^2^ = 0.12). The post-hoc tests revealed that the amplitude for neutral stimuli (*M* = 2.54, *SEM* = 0.71) was significantly less positive than both for the negative (*M* = 3.03, *SEM* = 0.71; *t*(33) = -2.43, *p* = .041, *d* = -0.85) and positive (*M* = 3.04, *SEM* = 0.74; *t*(33) = 3.16, *p* = .010, *d* = 1.10) stimuli (Fig 5J).

In the **time window of 390–550 ms**, we observed only a main effect of flanker congruency (*F*(1, 33) = 14.39, *p* = .001; *η*^*2*^ = 0.30). The amplitude was significantly more positive in the incongruent (*M* = 2.26, *SEM* = 0.72) than in the congruent condition (*M* = 1.80, *SEM* = 0.69; *t*(33) = 3.79, *p* = .001, *d* = 1.32). The main effects of the other factors were insignificant.

#### 3.2.2 Classical approach

Besides the exploratory approach, we also analysed relevant components known from the literature to possibly be related to the tasks in the current experiment. We analysed the mean amplitude in the EPN, P2, N450, and LPC components. In the following subsections, we describe the details of effects found for each of them. Additionally, in S2 Appendix we report the descriptive statistics and ANOVa results including the non-significant main effects.

**Fig 6:**
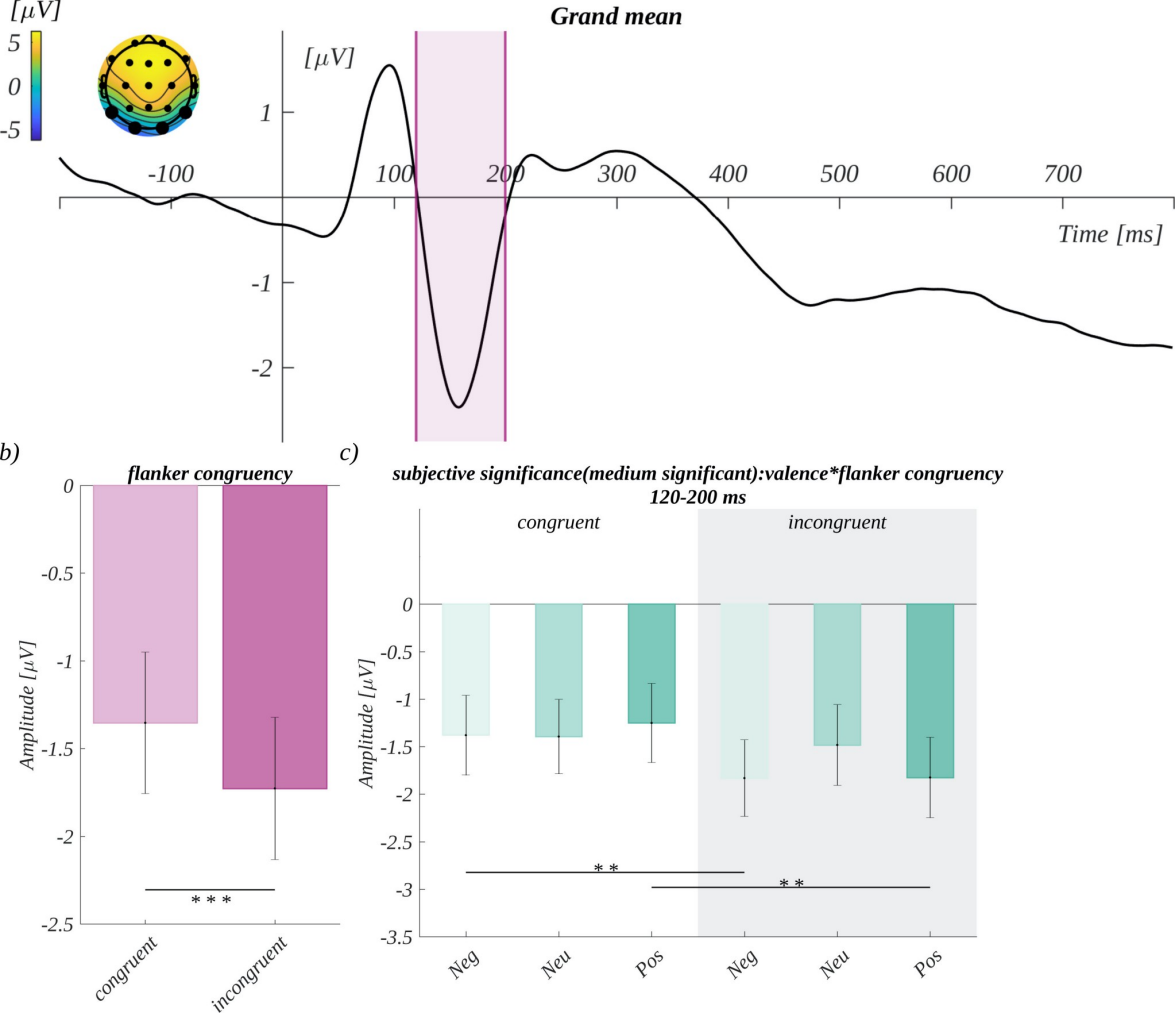
a) Grand mean for the EPN component. The time of the EPN waveform is marked in pink. In the inset, the topography of the mean amplitude is shown, the enlarged dots mark the channels constituting the ROI_EPN_. b) The main effect of flanker congruency. c) Effect of the interaction between valence and flanker congruency for a moderate level of subjective significance.

We analysed the mean amplitude in the time window 100 ms to 200 ms (Fig 6A) in the region of interest characteristic for the EPN component, ROI_EPN_, i.e., (O1, O2, T5, T6). We obtained the main effect of flanker congruency (*F*(1, 33) = 31.60, *p* < .001; *η*^*2*^ = 0.49) The amplitude for incongruent colours (*M* = -1.73, *SEM* = 0.41) was significantly more negative than for the congruent condition (*M* = -1.35, *SEM* = 0.40; *t*(33) = -5.62, *p* < .001, *d* = -1.96) (Fig 6B). The main effects of the other factors were insignificant.

Additionally, we observed an interaction between subjective significance, valence, and flanker congruency (*F*(4, 132) = 2.50, *p* = .045; *η*^*2*^ = 0.07). Further analysis within the levels of subjective significance showed that there was an interaction between valence and flanker congruency for moderately significant conditions (*F*(2, 66) = 4.92, *p* = .010; *η*^*2*^ = 0.13). The post-hoc tests revealed that the amplitude for negative incongruent stimuli (*M* = -1.83, *SEM* = 0.40) was significantly more negative than for the negative congruent stimuli (*M* = -1.38, *SEM* = 0.42; *t*(33) = -3.76, *p* = .009, *d* = -1.31) (Fig 6C). Furthermore, the amplitude in the positive incongruent condition (*M* = -1.82, *SEM* = 0.42) was significantly more negative than in the positive congruent condition (*M* = -1.25, *SEM* = 0.42; *t*(33) = -3.98, *p* = .005, *d* = -1.39) (Fig 6C).

**Fig 7:**
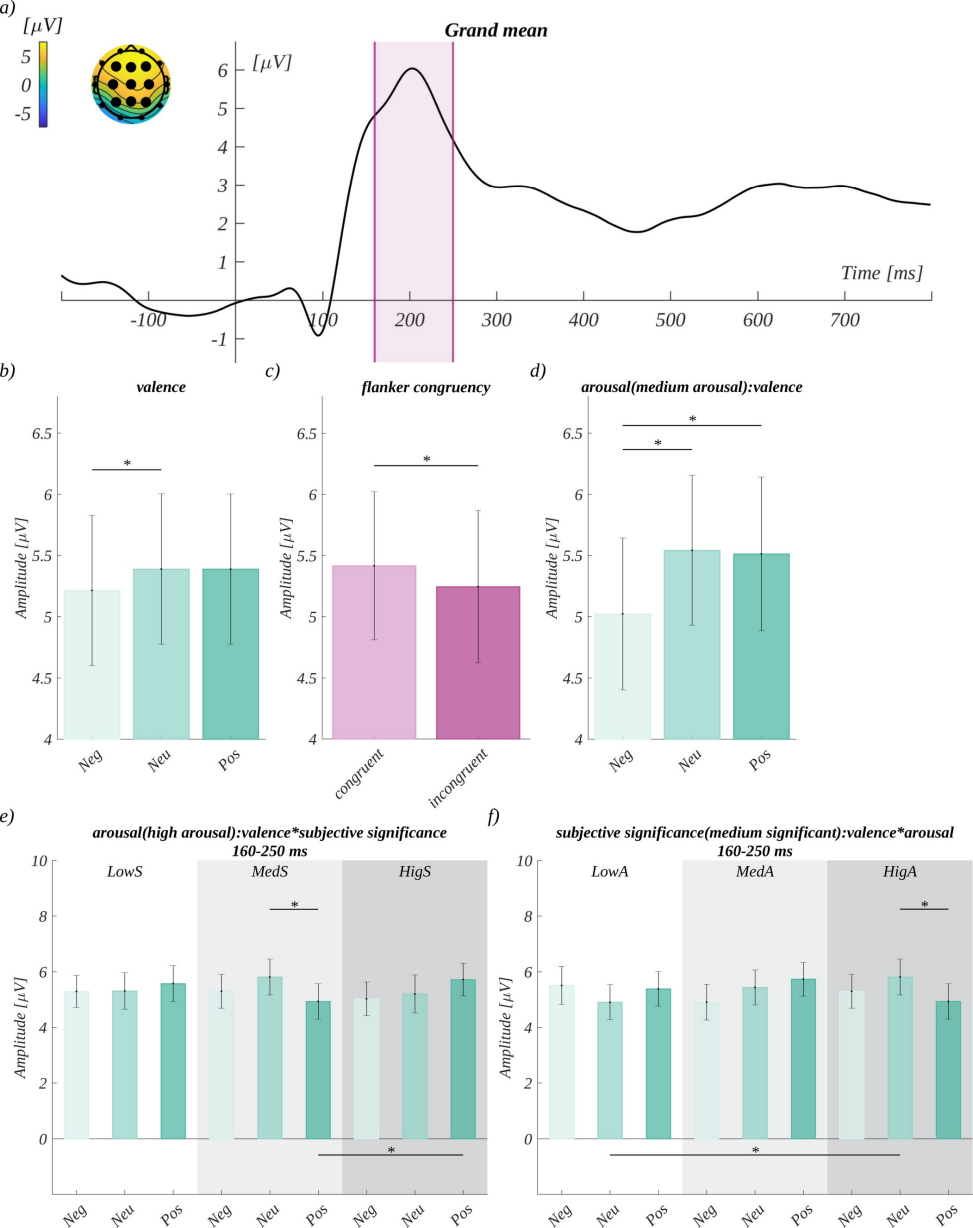
The effects found for the P2 component. a) Grand mean for the P2 component. The time of the P2 waveform is marked in pink. In the inset, the topography of the mean amplitude in this time window is shown; the enlarged dots mark the channels constituting the ROI_P2_. b) Main effect of valence. c) Main effect of flanker congruency. d) Effect of valence for moderate arousal words. e) Interaction between valence and subjective significance for high arousal words. f) Interaction between valence and arousal for moderately significant words.

We analysed the mean **P2** amplitude in the time window 160–250 ms in the region of interest characteristic for this component (ROI_P2_), i.e., F3, Fz, F4, C3, Cz, C4, P3, Pz, and P4. The ground mean for the P2 component is shown in Fig 7A. We observed the main effect of valence (*F*(2, 66) = 4.25, *p* = .018; *η*^*2*^ = 0.11). The post-hoc tests showed that the amplitude for neutral (*M* = 5.39, *SEM* = 0.61) was significantly more positive than for the negative condition (*M* = 5.21, *SEM* = 0.61; *t*(33) = 2.92, *p* = .019, *d* = 1.02) (Fig 7B). Furthermore, we obtained a main effect of flanker congruency (*F*(1, 33) = 5.35, *p* = .027; *η*^*2*^ = 0.14). The amplitude for the congruent condition (*M* = 5.42, *SEM* = 0.60) was more positive than for the incongruent one (*M* = 5.24, *SEM* = 0.62; *t(*33) = -2.31, *p* = .027, *d* = -0.81) (Fig 7C). The main effects of arousal and subjective significance were not insignificant.

Moreover, we observed an interaction between arousal and valence (*F*(3.12, 102.95) = 3.75, *p* = .012, *η*^*2*^ = 0.10). Mauchly’s test indicated that the assumption of sphericity had been violated for this factor (Xi2 (4) = 0.55, p = .027), so degrees of freedom were corrected using Greenhouse-Geisser estimates of sphericity (epsilon = 0.78). Further analysis showed that for moderate arousal words there was an effect of valence (*F*(2, 66) = 7.40, *p* = .001; *η*^*2*^ = 0.18). The post-hoc tests in this condition revealed that the amplitude for negative stimuli (*M* = 5.02, *SEM* = 0.62) was significantly less positive than for both neutral (*M* = 5.54, *SEM* = 0.61; *t*(33) = 3.32, *p* = .007, *d* = 1.16) and positive ones (*M* = 5.51, *SEM* = 0.63; *t*(33) = 2.89, *p* = .013, *d* = 1.01) (Fig 7D).

Furthermore, we obtained a three-way interaction between valence, arousal, and subjective significance (*F*(8, 264) = 4.12, *p* < .001; *η*^*2*^ = 0.11). We analysed it further with a series of two-way ANOVAs, each time keeping successive levels of one of the factors constant. The analysis within the levels of arousal showed that for the high arousal condition, there was an interaction between valence and subjective significance (*F*(4, 132) = 5.17, *p* = .001; *η*^*2*^ = 0.14). The post-hoc tests showed that, in the case of moderately significant words of neutral valence (*M* = 5.81, *SEM* = 0.65), the amplitude was significantly more positive than for words of positive valence (*M* = 4.93, *SEM* = 0.64; *t*(33) = -3.81, *p* = .020, *d* = -1.33). Moreover, for positively valenced words, the amplitude was significantly more positive in the condition of high subjective significance (*M* = 5.72, *SEM* = 0.58) than in the moderately subjective significant condition (*M* = 4.93, *SEM* = 0.64*; t*(33) = -3.54, *p* = .044, *d* = -1.23) (Fig 7E). For the neutral valence condition, we obtained an interaction of arousal and subjective significance (*F*(4, 132) = 3.01, *p* = .020; *η*^*2*^ = 0.08). The post-hoc tests showed that the amplitude for moderately significant and high arousal words (*M* = 5.81, *SEM* = 0.65) was more positive than for moderately significant but low arousal words (*M* = 4.90, *SEM* = 0.63; *t*(33) = -3.77, *p* = .023, *d* = -1.31) (Fig 7E).

**Fig 8:**
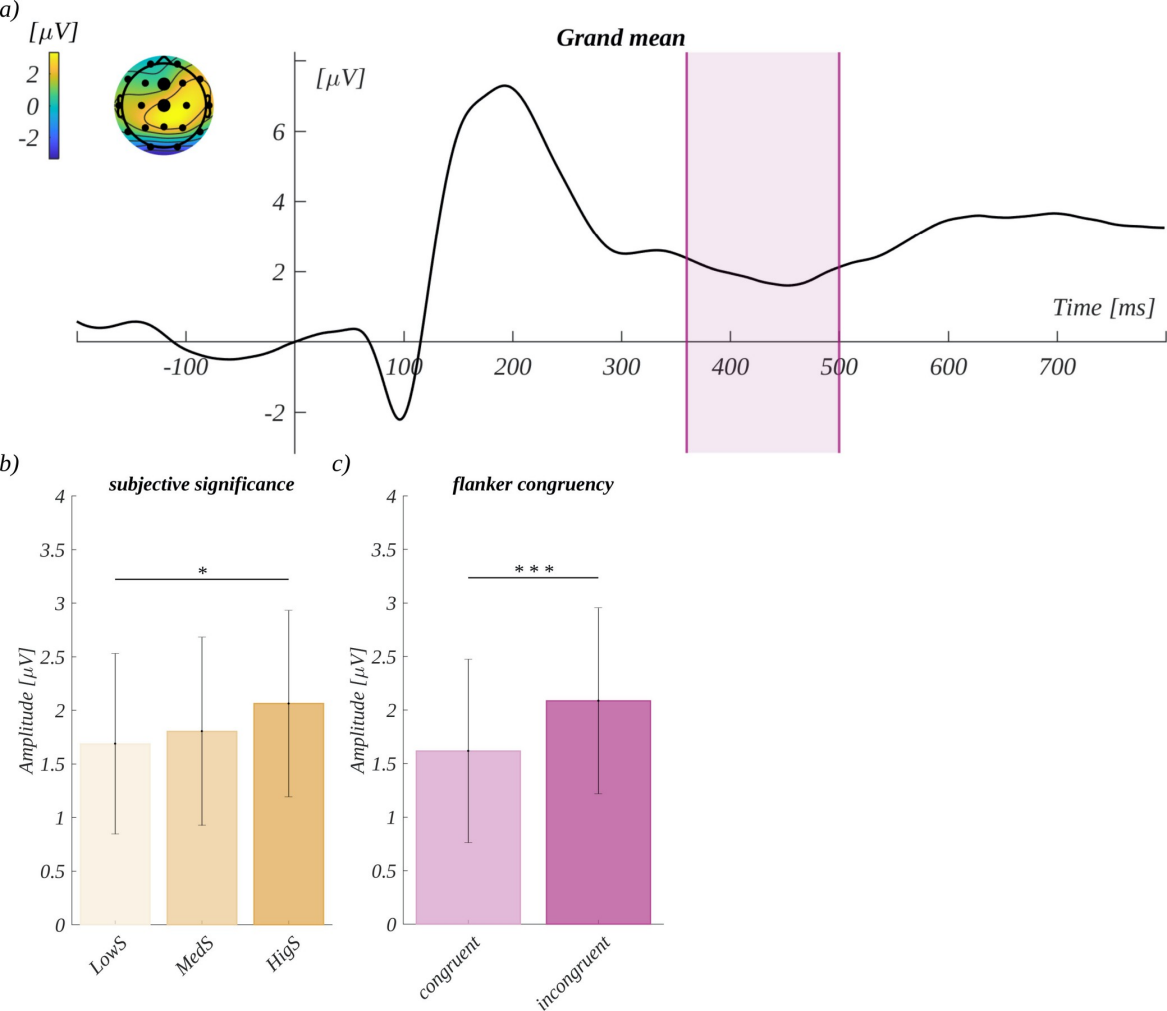
The effects found for the N450 component. a) The grand mean ERP for the Cz and Pz channels, the time of N450 is marked. The topography of the mean amplitude on the selected time range is shown in the inset. b) The main effect of subjective significance. c) The main effect of flanker congruency.

The time range for the **N450** component was adjusted to 360–500 ms. The mean amplitude for this component was averaged across the Cz and Pz channels (Fig 8). We observed a main effect of subjective significance *F*(2, 66) = 4.32, *p* = .017; *η*^*2*^ = 0.12). The amplitude for highly significant words (*M* = 2.06, *SEM* = 0.87) was more positive than for low significant stimuli (*M* = 1.69, *SEM* = 0.84; *t*(33) = -3.00, *p* = .015, *d* = -1.05) (Fig 8B). Furthermore, we observed the main effect of flanker congruency (*F*(1, 33) = 13.78, *p* = .001; *η*^*2*^ = 0.29). Here, the amplitude for incongruent condition (*M* = 2.09, *SEM* = 0.87) was significantly more positive than in the congruent condition (*M* = 1.62, *SEM* = 0.86; *t*(33) = 3.71, *p* = .001, *d* = 1.29). The main effects for the other two factors were not significant.

The amplitude of the **LPC** component was analysed in the time window from 450–800 ms in the region of interest characteristic for this component: P3, Pz, and P4. None of the main effects was significant, i.e., valence (*F*(2, 66) = 0.69, *p* = 0.50, *η*^*2*^ = 0.02), arousal (*F*(2, 66) = 0.24, *p* = 0.79, *η*^*2*^ = 0.01), subjective significance (*F*(2, 66) = 0.85, *p* = 0.43, *η*^*2*^ = 0.02), and flanker congruency (*F*(1, 33) = 0.81, *p* = 0.37, *η*^*2*^ = 0.02).

## 4. Discussion

This experiment was the first to search for the role of emotional factors such as valence arousal and subjective significance in the control of inhibition effectiveness, measured for both perceptual and conceptual control. We expected two distinct effects due to two types of inhibition. On the behavioural level, we observed them all simultaneously, but in ERPs, analyses differentiated perceptual inhibition from conceptual inhibition.

### 4.1. Behavioural results

At the behavioural level, the current study revealed a significant difference in reaction times between congruent and incongruent trials. Such an effect is consistent with the effect of congruency observed in both the classical Stroop task [37,40,41,113,127,128] and in studies employing the flanker task [29,37,38,40,41,57,116,129]. Taking these into account, we might conclude that the experiment’s paradigm employed interference control accordingly and thus delivers useful modification and synthesis of both paradigms of a flanker task and EST.

The second observed result was the effect of subjective significance; words low on this dimension elicited longer reaction times than those with moderate significance. This effect is partially congruent with previous studies employing the EST and combined Stroop task (with emotional words displayed below and above the colour-meaning word of the classical Stroop task), where mildly significant words evoked longer reaction times than highly significant ones [25,98]. Typically, such effects are because some stimuli are less commonly used. Thus, they require more time to be processed, but in the current experiment, words were aligned for the frequency of usage in the Polish language, so this explanation is not valid here. The effect observed in this study, together with those from previous studies, suggests that high subjective significance can speed up the reaction in tasks requiring cognitive control.

The last behavioural result was an interaction between valence and arousal. The arousal effect was found for neutrally valenced stimuli: reaction latencies to trials with high arousal stimuli were longer than both low and moderate arousal ones, which remains in line with the current state of knowledge about the influence of arousal on cognitive control [6,7,13,14,28,36,130–134]. A high load of arousal slows down reactions, as the words bring a high emotional charge to processing. In contrast to previous studies (e.g., [14,58,94,129,135,136]), we did not observe a valence effect in the current study. However, some studies have revealed a leading role of arousal in tasks employing cognitive control, when valence and arousal are precisely controlled in orthogonally crossed manipulations [57,114,116,119,127]⁠.

### 4.2. Electrophysiological results

We will discuss EEG results starting from the earliest effects after stimulus onset. In the exploratory analysis, we note that the perceptual effect of flanker incongruence started as early as 60 ms after onset. In the first 60–120 ms window, the amplitude for incongruent colours was more negative when compared to congruent colours. The effect of the flanker persisted throughout the analysis. However, from the second window (120–220 ms) onward, the direction of the effect flipped, as incongruent colours produced a more positive amplitude when compared to congruent colours. The effect of incongruent contextual information observed as an EPN manifestation was revealed experimentally [137]. This global effect may be seen as the reflection of a general slow-down in the processing of incongruent trials that was reflected in behavioural results. However, early after stimuli onset we also observed some more localized effects of incongruent colors, which indicated early preferential processing of conflict between possible responses in the frontal regions. Specifically, in the 120–220 ms time window, there was an interaction between ROIs and congruency. The post-hoc analysis indicated it stemmed not from a change in direction of the effect, but a larger size of the effect in the frontal compared to central electrodes. This result is congruent with previous research indicating that in such tasks, the resolution of flanker conflict is associated with components in the 120–220 ms time range [48]. This result expands this result by suggesting that this resolution may be performed primarily in the frontal regions.

The first component in the classical analysis was the EPN, a negative deflection of amplitude observed in occipito-temporal regions, commonly occurring between 200 and 300 ms after stimulus presentation [82]. However, our analysis indicated an EPN component occurring before 200 ms, that is, before the start of the first electrophysiological signs of processing of the word’s meaning [87]. We obtained a main effect of congruency; a higher amplitude was observed for the incongruent colour of a word than for the congruent condition.

Despite such an early EPN time range, we observed some emotional modulation: we obtained a third-order interaction between colour congruence, valence, and subjective significance. In the moderate significance condition, there was an interaction between valence and congruence. For both positive and negative valence, congruence conditions differed in amplitude in the same way as in the main effect (more positive amplitude for incongruent stimuli). This effect, however, disappeared in the neutral valence condition. Research has shown that a larger amplitude of EPN component occurs for emotionally charged words [83,100,105,138]. However, as this effect occurred before the analysis of the word’s meaning, so this effect cannot be attributed directly to emotional word processing. As emotional effects have repeatedly been reported to start only after 200ms [100], two possible explanations of this effect emerge. First, the effect could be a sign of some rapid decoding of the word’s emotional load. This is extremely unlikely as such rapid reactions seem to be possible only for high-frequency words, yet we controlled for frequency while picking word stimuli for this experiment. Such an effect would also likely not be a complex interaction like the one we observed. However, this effect becomes theoretically interesting when we consider that, as the present study employed a block design, a second explanation is more credible. Namely, that such early emotional interference should be interpreted as stemming from the affect induced in previous trials of the same levels of emotional factors, i.e., a spillover effect. The influence that the emotional load of the previous trial has on responses has been shown even on the level of behavioral effects [89]. Thus a subsequent presentation of many stimuli with the same emotional load has all the more power to influence early potentials, which presents a prime opportunity to observe the interaction between perceptual and emotional interference. Interpreting the effect in this light, we see that no congruence effect appears in blocks of sufficiently neutral emotional load (moderate significance, neutral valence). Thus, employing a crossover design with both emotional and perceptual interference allowed us to observe that the perceptual interference is exacerbated under affect induced from previous stimuli. Coming to the P2 component, we observed an apparent effect of incongruent colours of flanker words. This modulation continues from the modulation observed in the EPN and exhibits a pattern repeated throughout the flanker literature, in which congruent trials produce a greater amplitude than incongruent ones [42,44–46,48].

It is at this component that we started to observe a simultaneous, parallel occurrence of perceptual and emotional interference, indexed by the start of emotional modulation. Precisely, we observed a main effect of valence: neutral words produced a larger P2 amplitude than negative words. Moreover, we saw an interaction between valence and arousal. The shape of this interaction showed that the main effect of valence was exacerbated in the high arousal condition. There, both positive and neutral words produced larger P2 amplitudes than negative words. A valence effect was expected; the direction of differences, however, is different from that commonly reported [91–94]. Three key things may help us to understand this effect: behavioural results, task differences, and the role of subjective significance.

Firstly, this pattern may reflect the fact that no behavioural effect of valence was found. P2 amplitudes tend to resemble behavioural responses and serve as an even more sensitive measure of inhibitory control [99]. For this reason, even when a pattern of behavioural slow down for valenced words is not present, we may still observe emotional modulation. However, the direction of amplitude differences may be different, revealing the underlying cognitive control mechanism involved in the task [98]. Causally, the shape of the observed pattern may come down to the specifications of the task [82], as our task differs from previous EST studies by the inclusion of flanker words and the inclusion of subjective significance as a controlling factor. Suppose subjective significance is a factor that partly explains previously seen emotional effects. In that case, its inclusion may have contributed to a change in the contribution of valence to emotional interference, indexed by the lack of a behavioural effect of valence.

The third level interaction between valence, arousal, and subjective significance may give us a clue about the role that controlling arousal and subjective significance plays in the shape of this component. Notably, for this discussion, we observed that the amplitude for positive words significantly differed between levels of subjective significance for high arousal words, and the amplitude for neutral words differed between levels of arousal in the moderately subjective significance condition. As the amplitude for positive and neutral words, relative to negative words, is the source of the different patterns of valence effects, this points to a crucial role in controlling these factors and examining their interaction with each other. Crucially, this also suggests that all three factors in some regard contribute to modulating the processing of words and inhibitory control in this time window.

Interestingly, in the exploratory analysis, valence effects failed to reach significance in the two corresponding time windows of 120–220 ms and 220–290 ms. Only in the third window of 290–390 ms was there significant emotional modulation of the ERP amplitude. The factor that instigated this modulation, however, was subjective significance.

The N450 component is connected with conflict detection and conflict monitoring in interference control [139]. For this component, we observed a more positive amplitude for incongruent trials in comparison with congruent ones, which puts the present study in line with effects reported in the flanker literature [140]. Moreover, a subjective significance effect was observed, in that the amplitude for highly significant words was more positive than for words with a low score of this factor. This result indicates a clear difference in the processing of low and highly significant stimuli, pointing to the prominent role of this factor for cognitive control. As before, we noted the absence of an arousal effect; together, these results indicate that subjective significance may be a factor that better explains the previous effects of the emotional load of words seen in studies of control inhibition.

Comparing these results to the exploratory analysis, we see that the flanker effect, which started at around 60 ms, persists in the 390–550 ms window, as the amplitude was significantly more positive in the incongruent condition than in the congruent condition. However, there is no corresponding effect of subjective significance in this window. We may now see that the subjective significance modulation we had seen in the previous window was not associated with the emotional modulation of the P2 component, but with the modulation of N450. Thus, we may speculate that the process responsible for modulation in the N450 time window started even earlier, maybe as early as 290 ms after stimulus onset.

Lastly, we should discuss the lack of the predicted valence effect on the LPC. While early studies of emotional word processing reported emotional modulation of the LPC in tasks such as the EST [83,84,94,104], a difference between these and later studies, that could’ve caused this result may have been that these early studies frequently didn’t control for factors such as arousal. However, a more general principle may better explain the observed lack of LPC modulation. As the LPC is associated with later stages of semantic processing [105,106] and conscious attention to the word [107], our result may be seen as supporting the more recent proposition, that the LPC becomes more emotionally modulated, as the level of attention to the word’s emotionality increases [108,109], and thus that we should not expect the LPC to be emotionally modulated in tasks such as the EST, that does not draw the participants attention to the word’s meaning. Concretely, our result mirrors the mentioned results of González-Villar et al. [108], who compared the LPC modulation using the Emotional Stroop Task and the Emotional Decision Task and found it to be modulated by valence only in the latter, as well as corroborate the results of our previous EST study [99].

### 4.3. Limitations

There are several limitations of the study. The first one refers to the study design. Three dimensions were crossed orthogonally and used to create the study design: valence, arousal, and subjective significance. In general, affective dimensions are correlated to each other. For example, a U-shaped relation naturally occurs between valence and arousal, i.e., highly negative and positive stimuli are characterised by high arousal [20]. On the other hand, the advantages of the applied design are more significant than potential risks: the orthogonal design enables (1) the identification of effects that may work in opposite directions in the natural world (i.e., increasing arousal and increasing subjective significance levels), and (2) controlling for factors such as word length and frequency of usage in language.

Another limitation is related to sample selection. The study was run on a group of students. Although the sample was highly homogenous and specific, this type of sample selection allowed for omitting confounding factors related to the diversification of cognitive abilities according to age and education and was congruent with the sample assessing the word stimuli in affective norms studies used for selection of stimuli for an affective manipulation.

### 4.4. Conclusions

The current electrophysiological experiment was investigating the role of the three emotional factors simultaneously, namely valence, arousal, and subjective significance, in processes of inhibition control. The advantage of our approach was the use of orthogonal manipulation, providing the opportunity to precisely separate the effect of each emotional dimension on control effectiveness. We have also used a paradigm merging two types of interferences: perceptual (associated with physical traits interferences in vision) and conceptual (associated with meaning interferences). We have shown that: (1) to some extent, interferences at the perceptual and conceptual levels are distinct from each other, i.e., the flanker congruency effect (more polarised amplitude for the incongruent condition in comparison to congruent conditions) was present only in the EPN component, while in the later component, the effect was significant, but surprisingly reversed (more polarised amplitude for the congruent condition in comparison to incongruent conditions). (2) Emotional factors shape each interference type in a different way, i.e., valence was found to interact with earlier ERP components (EPN, P2), while subjective significance was found to interact with the later component (N450). (3) Once more [24,25,99,112,121,141–144], subjective significance was found to reduce cognitive control costs, both in behavioural measures and indexed by the amplitude of the N450 component.

## Supporting information

S1 Appendix The list of words used in the experiment and analises showing their validity as experimental stimuli. (XLSX)

S2 Appendix The descriptive statistics and ANOVa results including the non-significant main effects for EEG results. (XLSX)

## References

[1] May CP, Kane MJ, Hasher L. Determinants of Negative Priming. Psychol Bull. 1995;118: 35–54. doi: 10.1037/0033-2909.118.1.35 7644605

[2] Gratton G, Cooper P, Fabiani M, Carter CS, Karayanidis F. Dynamics of cognitive control: Theoretical bases, paradigms, and a view for the future. Psychophysiology. 2018;55: e13016. doi: 10.1111/psyp.13016 29044552

[3] Miyake A, Friedman NP. The nature and organization of individual differences in executive functions: Four general conclusions. Curr Dir Psychol Sci. 2012;21: 8–14. doi: 10.1177/0963721411429458 22773897 PMC3388901

[4] Nigg JT. On inhibition/disinhibition in developmental psychopathology: views from cognitive and personality psychology and a working inhibition taxonomy. Psychol Bull. 2000;126: 220–246. doi: 10.1037/0033-2909.126.2.220 10748641

[5] Eriksen BA, Eriksen CW. Effects of noise letters upon the identification of a target letter in a nonsearch task. Percept Psychophys. 1974;16: 143–149. doi: 10.3758/BF03203267

[6] Ben-Haim MS, Williams P, Howard Z, Mama Y, Eidels A, Algom D. The emotional stroop task: Assessing cognitive performance under exposure to emotional content. J Vis Exp. 2016;2016. doi: 10.3791/53720 27405091 PMC4993290

[7] Imbir KK, Pastwa M, Jankowska M, Kosman M, Modzelewska A, Wielgopolan A. Valence and arousal of words in visual and conceptual interference control efficiency. Zajenkowski M, editor. PLoS One. 2020;15: e0241694. doi: 10.1371/journal.pone.0241694 33211720 PMC7676691

[8] Russell JA. A circumplex model of affect. J Pers Soc Psychol. 1980;39: 1161–1178. doi: 10.1037/h0077714

[9] Barrett LF. Discrete Emotions or Dimensions? The Role of Valence Focus and Arousal Focus. Cogn Emot. 1998;12: 579–599. doi: 10.1080/026999398379574

[10] Lazarus RS. Cognition and motivation in emotion. Am Psychol. 1991;46: 352–367. doi: 10.1037//0003-066x.46.4.352 2048794

[11] Zajonc RB. Feeling and Thinking: Preferences Need No Inferences. Am Psychol. 1980;35: 1S1. doi: 10.1634/theoncologist.9-90005-10 15591418

[12] Damasio A. Self comes to mind: constructing the conscious mind. New York, NY: Pantheon; 2010.

[13] Dresler T, Mériau K, Heekeren HR, Meer E. Emotional Stroop task: effect of word arousal and subject anxiety on emotional interference. Psychol Res Psychol Forsch. 2009;73: 364–371. doi: 10.1007/s00426-008-0154-6 18636272

[14] Demanet J, Liefooghe B, Verbruggen F. Valence, arousal, and cognitive control: A voluntary task-switching study. Front Psychol. 2011;2. doi: 10.3389/fpsyg.2011.00002 22131982 PMC3223383

[15] Ding J, Wang L, Yang Y. The dynamic influence of emotional words on sentence comprehension: An ERP study. Cogn Affect Behav Neurosci. 2016;16: 433–446. doi: 10.3758/s13415-016-0403-x 26833049

[16] Kuperman V. Virtual experiments in megastudies: A case study of language and emotion. Q J Exp Psychol. 2015;68: 1693–1710. doi: 10.1080/17470218.2014.989865 25406972 PMC4494982

[17] Fox E, Russo R, Bowles R, Dutton K. Do threatening stimuli draw or hold visual attention in subclinical anxiety? J Exp Psychol Gen. 2001;130: 681–700. doi: 10.1037/0096-3445.130.4.681 11757875 PMC1924776

[18] Ohman A, Mineka S. Fears, phobias, and preparedness: toward an evolved module of fear and fear learning. Psychol Rev. 2001;108: 483–522. doi: 10.1037/0033-295x.108.3.483 11488376

[19] Epstein S. Cognitive-Experiential Self-Theory of Personality. Handbook of Psychology. 2003. doi: 10.1002/0471264385.wei0507

[20] Imbir K. From heart to mind and back again. A duality of emotion overview on emotion-cognition interactions. New Ideas Psychol. 2016;43: 39–49. doi: 10.1016/j.newideapsych.2016.04.001

[21] Bradley M, Lang P. Affective Norms for English Words (ANEW): Stimuli, instruction manual and affective ratings (Technical Report No. C-1). University of Florida, Center for Research in Psychophysiology; 1999.

[22] Montefinese M, Ambrosini E, Fairfield B, Mammarella N. The adaptation of the Affective Norms for English Words (ANEW) for Italian. Behav Res Methods. 2014;46: 887–903. doi: 10.3758/s13428-013-0405-3 24150921

[23] Wierzba M, Riegel M, Pucz A, Leśniewska Z, Dragan WŁ, Gola M, et al. Erotic subset for the Nencki Affective Picture System (NAPS ERO): cross-sexual comparison study. Front Psychol. 2015;6. doi: 10.3389/fpsyg.2015.00006 26441715 PMC4564755

[24] Imbir K, Jarymowicz M. The effect of automatic vs. Refl ective emotions on cognitive control in antisaccade tasks and the emotional stroop test. Polish Psychol Bull. 2013;44. doi: 10.2478/ppb-2013-0016

[25] Imbir K. Subjective Significance Shapes Arousal Effects on Modified Stroop Task Performance: A Duality of Activation Mechanisms Account. Front Psychol. 2016;7: 75. doi: 10.3389/fpsyg.2016.00075 26869974 PMC4735373

[26] Baumeister RF, Bratslavsky E, Muraven M, Tice DM. Ego depletion: is the active self a limited resource? J Pers Soc Psychol. 1998;74: 1252–1265. doi: 10.1037//0022-3514.74.5.1252 9599441

[27] Kahnt T, Tobler PN. Salience Signals in the Right Temporoparietal Junction Facilitate Value-Based Decisions. J Neurosci. 2013;33: 863–869. doi: 10.1523/JNEUROSCI.3531-12.2013 23325225 PMC6704859

[28] Ashley V, Swick D. Consequences of emotional stimuli: Age differences on pure and mixed blocks of the emotional Stroop. Behav Brain Funct. 2009;5: 14. doi: 10.1186/1744-9081-5-14 19254381 PMC2661089

[29] Rowe G, Hirsh JB, Anderson AK. Positive affect increases the breadth of attentional selection. Proc Natl Acad Sci U S A. 2007;104: 383–388. doi: 10.1073/pnas.0605198104 17182749 PMC1765470

[30] Fenske MJ, Eastwood JD. Modulation of Focused Attention by Faces Expressing Emotion: Evidence from Flanker Tasks. Emotion. 2003;3: 327–343. doi: 10.1037/1528-3542.3.4.327 14674827

[31] Horstmann G, Scharlau I, Ansorge U. More efficient rejection of happy than of angry face distractors in visual search. Psychon Bull Rev. 2006;13: 1067–1073. doi: 10.3758/bf03213927 17484437

[32] Zetsche U, Joormann J. Components of interference control predict depressive symptoms and rumination cross-sectionally and at six months follow-up. J Behav Ther Exp Psychiatry. 2011;42: 65–73. doi: 10.1016/j.jbtep.2010.06.001 20650447

[33] van Steenbergen H, Band GPH, Hommel B. In the Mood for Adaptation. Psychol Sci. 2010;21: 1629–1634. doi: 10.1177/0956797610385951 20943936

[34] Finkenzeller T, Würth S, Doppelmayr M, Amesberger G. Temporal Dynamics of Varying Physical Loads on Speed and Accuracy of Cognitive Control. J Sport Exerc Psychol. 2019;41: 206–214. doi: 10.1123/jsep.2018-0239 31427542

[35] Imbir K. Valence and Origin of Emotional Words Influence on Subsequent Perception of Ambiguous Stimuli in Terms of Competence Versus Warmth. J Psycholinguist Res. 2017; 1–10. doi: 10.1007/s10936-016-9417-3 28639175 PMC5655634

[36] Imbir K. Words’ Origin of an Affective State, but not Valence, Shape the Reaction Latencies in a Word-Sign Choosing Ambiguous Task. Current Psychology. 2017: 1–10. doi: 10.1007/s12144-017-9669-6

[37] Verbruggen F, Liefooghe B, Vandierendonck A. The interaction between stop signal inhibition and distractor interference in the flanker and Stroop task. Acta Psychol (Amst). 2004;116: 21–37. doi: 10.1016/j.actpsy.2003.12.011 15111228

[38] Bruyneel L, van Steenbergen H, Hommel B, Band GPH, De Raedt R, Koster EHW. Happy but still focused: Failures to find evidence for a mood-induced widening of visual attention. Psychol Res. 2013;77: 320–332. doi: 10.1007/s00426-012-0432-1 22466376

[39] Imbir K. Does Reading Words Differing in Arousal Load Influence Interference Control in Flanker Task? Curr Psychol. 2017;36: 157–166. doi: 10.1007/s12144-015-9396-9 28298876 PMC5329098

[40] Tillman CM, Wiens S. Behavioral and ERP indices of response conflict in Stroop and flanker tasks. Psychophysiology. 2011;48: 1405–1411. doi: 10.1111/j.1469-8986.2011.01203.x 21457276

[41] van Steenbergen H, Langeslag SJE, Band GPH, Hommel B. Reduced cognitive control in passionate lovers. Motiv Emot. 2014;38: 444–450. doi: 10.1007/s11031-013-9380-3

[42] Xie L, Ren M, Cao B, Li F. Distinct brain responses to different inhibitions: Evidence from a modified Flanker Task. Sci Rep. 2017;7: 1–10. doi: 10.1038/s41598-016-0028-x 28751739 PMC5532368

[43] Zhang J, Wu C, Yuan Z, Meng Y. Differentiating emotion-label words and emotion-laden words in emotion conflict: an ERP study. Exp Brain Res. 2019;237: 2423–2430. doi: 10.1007/s00221-019-05600-4 31302735

[44] Kałamała P, Szewczyk J, Senderecka M, Wodniecka Z. Flanker task with equiprobable congruent and incongruent conditions does not elicit the conflict N2. Psychophysiology. 2018;55: e12980. doi: 10.1111/psyp.12980 28845513

[45] Johnstone SJ, Barry RJ, Markovska V, Dimoska A, Clarke AR. Response inhibition and interference control in children with AD/HD: A visual ERP investigation. Int J Psychophysiol. 2009;72: 145–153. doi: 10.1016/j.ijpsycho.2008.11.007 19095016

[46] Jonkman LM, Kemner C, Verbaten MN, Engeland H, Kenemans JL, Camfferman G, et al. Perceptual and response interference in children with attention-deficit hyperactivity disorder, and the effects of methylphenidate. Psychophysiology. 1999;36: 419–429. doi: 10.1111/1469-8986.3640419 10432791

[47] Xie Y, Xu Y, Bian C, Li M. Semantic congruent audiovisual integration during the encoding stage of working memory: An ERP and sLORETA study. Sci Rep. 2017;7: 1–10. doi: 10.1038/s41598-016-0028-x 28698594 PMC5505990

[48] Rey-Mermet A, Gade M, Steinhauser M. Sequential conflict resolution under multiple concurrent conflicts: An ERP study. Neuroimage. 2019;188: 411–418. doi: 10.1016/j.neuroimage.2018.12.031 30562575

[49] Schmitt BM, Munte TF, Kutas M. Electrophysiological estimates of the time course of semantic and phonological encoding during implicit picture naming. Psychophysiology. 2000;37: 473–484. doi: 10.1111/1469-8986.3740473 10934906

[50] Olichney JM, Hansen LA, Hofstetter CR, Lee JH, Katzman R, Thal LJ. Association between severe cerebral amyloid angiopathy and cerebrovascular lesions in Alzheimer disease is not a spurious one attributable to apolipoprotein E4. Arch Neurol. 2000;57: 869–874. doi: 10.1001/archneur.57.6.869 10867785

[51] Appelbaum LG, Smith D V., Boehler CN, Chen WD, Woldorff MG. Rapid modulation of sensory processing induced by stimulus conflict. J Cogn Neurosci. 2011;23: 2620–2628. doi: 10.1162/jocn.2010.21575 20849233 PMC3096678

[52] McKay CC, van den Berg B, Woldorff MG. Neural cascade of conflict processing: Not just time-on-task. Neuropsychologia. 2017;96: 184–191. doi: 10.1016/j.neuropsychologia.2016.12.022 28017818 PMC5365079

[53] Donohue SE, Appelbaum LG, McKay CC, Woldorff MG. The neural dynamics of stimulus and response conflict processing as a function of response complexity and task demands. Neuropsychologia. 2016;84: 14–28. doi: 10.1016/j.neuropsychologia.2016.01.035 26827917 PMC4808442

[54] Finnigan S, Humphreys MS, Dennis S, Geffen G. ERP “old/new” effects: Memory strength and decisional factor(s). Neuropsychologia. 2002;40: 2288–2304. doi: 10.1016/s0028-3932(02)00113-6 12417459

[55] Olichney JM. Word repetition in amnesia: Electrophysiological measures of impaired and spared memory. Brain. 2000;123: 1948–1963. doi: 10.1093/brain/123.9.1948 10960058

[56] Larson MJ, Steffen PR, Primosch M. The impact of a brief mindfulness meditation intervention on cognitive control and error-related performance monitoring. Front Hum Neurosci. 2013;7: 308. doi: 10.3389/fnhum.2013.00308 23847491 PMC3705166

[57] Straub E, Kiesel A, Dignath D. Cognitive control of emotional distraction–valence-specific or general? Cogn Emot. 2019; 1–15. doi: 10.1080/02699931.2019.1666799 31532303

[58] Cohen N, Henik A, Moyal N. Executive control attenuates emotional effects-For high reappraisers only? Emotion. 2012;12: 970–979. doi: 10.1037/a0026890 22251044

[59] Mujica-Parodi L, Greenbag LR, Kilpatrick T. A Multi-Modal Study of Cognitive Processing under Negative Emotional Arousal. Proc Annu Meet Cogn Sci Soc. 2004;26: 26.

[60] Weinbach N, Kalanthroff E, Avnit A, Henik A. Can arousal modulate response inhibition? J Exp Psychol Learn Mem Cogn. 2015;41: 1873–1877. doi: 10.1037/xlm0000118 25867610

[61] Weinbach N, Henik A. The interaction between alerting and executive control: Dissociating phasic arousal and temporal expectancy. Attention, Perception, Psychophys. 2013;75: 1374–1381. doi: 10.3758/s13414-013-0501-6 23812913

[62] Larson MJ, Perlstein WM, Stigge-Kaufman D, Kelly KG, Dotson VM. Affective context-induced modulation of the error-related negativity. Neuroreport. 2006;17: 329–333. doi: 10.1097/01.wnr.0000199461.01542.db 16462607

[63] Hengstler M, Holland RW, Van Steenbergen H, Van Knippenberg A. The influence of approach-avoidance motivational orientation on conflict adaptation. Cogn Affect Behav Neurosci. 2014;14: 548–560. doi: 10.3758/s13415-014-0295-6 24841080

[64] Cromheeke S, Mueller SC. Probing emotional influences on cognitive control: An ALE meta-analysis of cognition emotion interactions. Brain Struct Funct. 2014;219: 995–1008. doi: 10.1007/s00429-013-0549-z 23563751

[65] McLean SP, Garza JP, Wiebe SA, Dodd MD, Smith KB, Hibbing JR, et al. Applying the Flanker Task to Political Psychology: A Research Note. Polit Psychol. 2014;35: 831–840. doi: 10.1111/pops.12056

[66] Alguacil S, Tudela P, Ruz M. Cognitive and affective control in a flanker word task: Common and dissociable brain mechanisms. Neuropsychologia. 2013;51: 1663–1672. doi: 10.1016/j.neuropsychologia.2013.05.020 23747603

[67] Fischer R, Schubert T. Valence processing bypassing the response selection bottleneck? Evidence from the psychological refractory period paradigm. Exp Psychol. 2008;55: 203–211. doi: 10.1027/1618-3169.55.3.203 18549168

[68] Pe ML, Vandekerckhove J, Kuppens P. A diffusion model account of the relationship between the emotional flanker task and rumination and depression. Emotion. 2013;13: 739–747. doi: 10.1037/a0031628 23527499

[69] Moser JS, Huppert JD, Duval E, Simons RF. Face processing biases in social anxiety: An electrophysiological study. Biol Psychol. 2008;78: 93–103. doi: 10.1016/j.biopsycho.2008.01.005 18353522

[70] Taylor DL, Grant DMM, Frosio KE, Kraft JD, Nagel KM, Deros DE, et al. Neural indices of orienting, discrimination, and conflict monitoring after contextual fear and safety learning. Cogn Affect Behav Neurosci. 2020;20: 917–927. doi: 10.3758/s13415-020-00810-8 32720204

[71] Dickter CL, Bartholow BD. Racial ingroup and outgroup attention biases revealed by event-related brain potentials. Soc Cogn Affect Neurosci. 2007;2: 189–198. doi: 10.1093/scan/nsm012 18985140 PMC2569810

[72] Denefrio S, Myruski S, Mennin D, Dennis-Tiwary TA. When neutral is not neutral: Neurophysiological evidence for reduced discrimination between aversive and non-aversive information in generalized anxiety disorder. Motiv Emot. 2019;43: 325–338. doi: 10.1007/s11031-018-9732-0 31105360 PMC6521852

[73] Stroop JR. Studies of interference in serial verbal reactions. J Exp Psychol. 1935;18: 643–662. doi: 10.1037/h0054651

[74] Larsen RJ, Mercer KA, Balota DA. Lexical characteristics of words used in emotional Stroop experiments. Emotion. 2006;6: 62–72. doi: 10.1037/1528-3542.6.1.62 16637750

[75] Burt JS. Why do non-color words interfere with color naming? J Exp Psychol Hum Percept Perform. 2002;28: 1019–1038. doi: 10.1037//0096-1523.28.5.1019 12421053

[76] Feng C, Becker B, Huang W, Wu X, Eickhoff SB, Chen T. Neural substrates of the emotion-word and emotional counting Stroop tasks in healthy and clinical populations: A meta-analysis of functional brain imaging studies. Neuroimage. 2018;173: 258–274. doi: 10.1016/j.neuroimage.2018.02.023 29496613

[77] Dalgleish T. Performance on the emotional stroop task in groups of anxious, expert, and control subjects: A comparison of computer and card presentation formats. Cogn Emot. 1995;9: 341–362. doi: 10.1080/02699939508408971

[78] Compton RJ, Banich MT, Mohanty A, Milham MP, Herrington J, Miller GA, et al. Paying attention to emotion: An fMRI investigation of cognitive and emotional Stroop tasks. Cogn Affect Behav Neurosci. 2003. doi: 10.3758/cabn.3.2.81 12943324

[79] Zhukova MA, Ovchinnikova I V, An I, Grigorenko EL. Altered neural processing of emotional words in adults with a history of institutionalization: Evidence from the emotional Stroop task. European Journal of Neuroscience; 2020. doi: 10.1111/ejn.15015 33080077

[80] Chung Y, Jeglic EL. (August 2017). “Detecting Suicide Risk Among College Students: A Test of the Predictive Validity of the Modified Emotional Stroop Task.” Suicide Life-Threatening Behav. 47: 398–409. doi: 10.1111/sltb.12287 27658610

[81] Palazova M, Sommer W, Schacht A. Interplay of emotional valence and concreteness in word processing: An event-related potential study with verbs. Brain Lang. 2013;125: 264–271. doi: 10.1016/j.bandl.2013.02.008 23578815

[82] Citron FMM. Neural correlates of written emotion word processing: A review of recent electrophysiological and hemodynamic neuroimaging studies. Brain Lang. 2012;122: 211–226. doi: 10.1016/j.bandl.2011.12.007 22277309

[83] Kissler J, Herbert C, Peyk P, Junghofer M. Early Cortical Responses to Emotional Words During Reading. Psychol Sci. 2007;18: 475–480. doi: 10.1111/j.1467-9280.2007.01924.x 17576257

[84] Schacht A, Sommer W. Time course and task dependence of emotion effects in word processing. Cogn Affect Behav Neurosci. 2009;9: 28–43. doi: 10.3758/CABN.9.1.28 19246325

[85] Kanske P, Kotz SA. Concreteness in emotional words: ERP evidence from a hemifield study. Brain Res. 2007;1148: 138–148. doi: 10.1016/j.brainres.2007.02.044 17391654

[86] Bayer M, Sommer W, Schacht A. P1 and beyond: Functional separation of multiple emotion effects in word recognition. Psychophysiology. 2012;49: 959–969. doi: 10.1111/j.1469-8986.2012.01381.x 22594767

[87] Nobre AC, Allison T, McCarthy G. Word recognition in the human inferior temporal lobe. Nature. 1994;372: 260–263. doi: 10.1038/372260a0 7969469

[88] Schacht A, Adler N, Chen P, Guo T, Sommer W. Association with positive outcome induces early effects in event-related brain potentials. Biol Psychol. 2012;89: 130–136. doi: 10.1016/j.biopsycho.2011.10.001 22027086

[89] McKenna FP, Sharma D. Reversing the Emotional Stroop Effect Reveals That It Is Not What It Seems: The Role of Fast and Slow Components. J Exp Psychol Learn Mem Cogn. 2004;30: 382–392. doi: 10.1037/0278-7393.30.2.382 14979812

[90] Huang Y-X, Luo Y-J. Temporal course of emotional negativity bias: An ERP study. Neurosci Lett. 2006;398: 91–96. doi: 10.1016/j.neulet.2005.12.074 16446031

[91] Schapkin SA, Gusev AN, Kühl J. Categorization of unilaterally presented emotional words: An ERP analysis. Acta Neurobiol Exp (Wars). 2000;60: 17–28. 10769926 10.55782/ane-2000-1321

[92] Carretié L, Hinojosa JA, Martín-Loeches M, Mercado F, Tapia M. Automatic attention to emotional stimuli: Neural correlates. Hum Brain Mapp. 2004;22: 290–299. doi: 10.1002/hbm.20037 15202107 PMC6871850

[93] Herbert C, Kissler J, Junghofer M, Peyk P, Rockstroh B. Processing of emotional adjectives: Evidence from startle EMG and ERPs. Psychophysiology. 2006;43: 197–206. doi: 10.1111/j.1469-8986.2006.00385.x 16712590

[94] van Hooff JC, Dietz KC, Sharma D, Bowman H. Neural correlates of intrusion of emotion words in a modified Stroop task. Int J Psychophysiol. 2008;67: 23–34. doi: 10.1016/j.ijpsycho.2007.09.002 17967496

[95] West R, Alain C. Effects of task context and fluctuations of attention on neural activity supporting performance of the Stroop task. Brain Res. 2000;873: 102–111. doi: 10.1016/s0006-8993(00)02530-0 10915815

[96] West R, Alain C. Event-related neural activity associated with the Stroop task. Cogn Brain Res. 1999;8: 157–164. doi: 10.1016/s0926-6410(99)00017-8 10407204

[97] West R, Bowry R, McConville C. Sensitivity of medial frontal cortex to response and nonresponse conflict. Psychophysiology. 2004;41: 739–748. doi: 10.1111/j.1469-8986.2004.00205.x 15318880

[98] Imbir K, Spustek T, Bernatowicz G, Duda J, Żygierewicz J. Two aspects of activation: Arousal and subjective significance—Behavioral and event-related potential correlates investigated by means of a modified emotional stroop task. Front Hum Neurosci. 2017;11. doi: 10.3389/fnhum.2017.00011 29311872 PMC5732992

[99] Thomas SJ, Johnstone SJ, Gonsalvez CJ. Event-related potentials during an emotional Stroop task. Int J Psychophysiol. 2007;63: 221–231. doi: 10.1016/j.ijpsycho.2006.10.002 17123655

[100] Herbert C, Junghofer M, Kissler J. Event related potentials to emotional adjectives during reading. Psychophysiology. 2008;45: 487–498. doi: 10.1111/j.1469-8986.2007.00638.x 18221445

[101] Cuthbert BN, Schupp HT, Bradley MM, Birbaumer N, Lang PJ. Brain potentials in affective picture processing: Covariation with autonomic arousal and affective report. Biol Psychol. 2000;52: 95–111. doi: 10.1016/s0301-0511(99)00044-7 10699350

[102] Hofmann MJ, Kuchinke L, Tamm S, Võ MLH, Jacobs AM. Affective processing within 1/10th of a second: High arousal is necessary for early facilitative processing of negative but not positive words. Cogn Affect Behav Neurosci. 2009;9: 389–397. doi: 10.3758/9.4.389 19897792

[103] Gootjes L, Coppens LC, Zwaan RA, Franken IHA, Van Strien JW. Effects of recent word exposure on emotion-word Stroop interference: An ERP study. Int J Psychophysiol. 2011;79: 356–363. doi: 10.1016/j.ijpsycho.2010.12.003 21156188

[104] Sass SM, Heller W, Stewart JL, Silton RL, Edgar JC, Fisher JE, et al. Time course of attentional bias in anxiety: emotion and gender specificity. Psychophysiology. 2010;47: 247–59. doi: 10.1111/j.1469-8986.2009.00926.x 19863758 PMC3073148

[105] Zhang D, He W, Wang T, Luo W, Zhu X, Gu R, et al. Three stages of emotional word processing: an ERP study with rapid serial visual presentation. Soc Cogn Affect Neurosci. 2014; 1–7. doi: 10.1093/scan/nst188 24526185 PMC4249467

[106] Hajcak G, MacNamara A, Olvet DM. Event-related potentials, emotion, and emotion regulation: an integrative review. Dev Neuropsychol. 2010;35: 129–155. doi: 10.1080/87565640903526504 20390599

[107] González-Villar AJ, Triñanes Y, Zurrón M, Carrillo-de-la-Peña MT. Brain processing of task-relevant and task-irrelevant emotional words: An ERP study. Cogn Affect Behav Neurosci. 2014;14: 939–950. doi: 10.3758/s13415-013-0247-6 24481851

[108] Hinojosa JA, Méndez-Bértolo C, Pozo MA. Looking at emotional words is not the same as reading emotional words: Behavioral and neural correlates. Psychophysiology. 2010;47: 748–757. doi: 10.1111/j.1469-8986.2010.00982.x 20158677

[109] Delaney-Busch N, Wilkie G, Kuperberg G. Vivid: How valence and arousal influence word processing under different task demands. Cogn Affect Behav Neurosci. 2016;16. doi: 10.3758/s13415-016-0402-y 26833048 PMC4870106

[110] Herbert C, Herbert BM, Ethofer T, Pauli P. His or mine? The time course of self-other discrimination in emotion processing. Soc Neurosci. 2011;6: 277–288. doi: 10.1080/17470919.2010.523543 21104542

[111] Imbir KK, Duda-Goławska J, Pastwa M, Jankowska M, Modzelewska A, Sobieszek A, et al. Electrophysiological and Behavioral Correlates of Valence, Arousal and Subjective Significance in the Lexical Decision Task. Front Hum Neurosci. 2020;14. doi: 10.3389/fnhum.2020.00014 33132881 PMC7575925

[112] Kanske P, Kotz SA. Leipzig Affective Norms for German: A reliability study. Behav Res Methods. 2010;42: 987–991. doi: 10.3758/BRM.42.4.987 21139165

[113] Feroz FS, Leicht G, Steinmann S, Andreou C, Mulert C. The Time Course of Activity within the Dorsal and Rostral-Ventral Anterior Cingulate Cortex in the Emotional Stroop Task. Brain Topogr. 2017. doi: 10.1007/s10548-016-0521-3 27659288

[114] Kanske P, Kotz SA. Positive emotion speeds up conflict processing: ERP responses in an auditory Simon task. Biol Psychol. 2011;87: 122–127. doi: 10.1016/j.biopsycho.2011.02.018 21382438

[115] Kanske P, Kotz SA. Effortful control, depression, and anxiety correlate with the influence of emotion on executive attentional control. Biol Psychol. 2012;91: 88–95. doi: 10.1016/j.biopsycho.2012.04.007 22564476

[116] Zeng Q, Qi S, Li M, Yao S, Ding C, Yang D. Enhanced conflict-driven cognitive control by emotional arousal, not by valence. Cogn Emot. 2017;31: 1083–1096. doi: 10.1080/02699931.2016.1189882 27249308

[117] Landman LL, van Steenbergen H. Emotion and conflict adaptation: the role of phasic arousal and self-relevance. Cogn Emot. 2020;34: 1083–1096. doi: 10.1080/02699931.2020.1722615 32036746

[118] Kanske P, Kotz SA. Modulation of early conflict processing: N200 responses to emotional words in a flanker task. Neuropsychologia. 2010;48: 3661–3664. doi: 10.1016/j.neuropsychologia.2010.07.021 20654636

[119] Kanske P, Kotz SA. Emotion Speeds up Conflict Resolution: A New Role for the Ventral Anterior Cingulate Cortex? Cereb Cortex. 2011;21: 911–919. doi: 10.1093/cercor/bhq157 20732901

[120] Imbir K. Affective Norms for 4900 Polish Words Reload (ANPW_R): Assessments for valence, arousal, dominance, origin, significance, concreteness, imageability and, age of acquisition. Front Psychol. 2016;7: 1081. doi: 10.3389/fpsyg.2016.01081 27486423 PMC4947584

[121] Lang PJ. Self-assessment manikin. Gainesville: FL: University of Florida; 1980.

[122] Kazojć J. Słownik frekwencyjny języka polskiego[Polish language dictionary of attendance]. 2011.

[123] Delorme A, Makeig S. EEGLAB: An open source toolbox for analysis of single-trial EEG dynamics including independent component analysis. J Neurosci Methods. 2004;134: 9–21. doi: 10.1016/j.jneumeth.2003.10.009 15102499

[124] Winkler I, Brandl S, Horn F, Waldburger E, Allefeld C, Tangermann M. Robust artifactual independent component classification for BCI practitioners. J Neural Eng. 2014;11: 035013. doi: 10.1088/1741-2560/11/3/035013 24836294

[125] Holm S. A simple sequentially rejective multiple test procedure. Scand J Stat. 1979.

[126] R Core Team. R: A language and environment for statistical computing. R Foundation for Statistical Computing. Vienna, Austria; 2017.

[127] Chajut E, Schupak A, Algom D. Emotional Dilution of the Stroop Effect: A New Tool for Assessing Attention Under Emotion. Emotion. 2010;10: 944–948. doi: 10.1037/a0020172 21058847

[128] Liotti M, Woldorff MG, Perez R, Mayberg HS. An ERP study of the temporal course of the Stroop color-word interference effect. Neuropsychologia. 2000;38: 701–711. doi: 10.1016/s0028-3932(99)00106-2 10689046

[129] Cohen N, Moyal N, Lichtenstein-Vidne L, Henik A. Explicit vs. implicit emotional processing: The interaction between processing type and executive control. Cogn Emot. 2016;30: 325–339. doi: 10.1080/02699931.2014.1000830 25621819

[130] Sharma D, McKenna FP. The role of time pressure on the emotional Stroop task. Br J Psychol. 2001. doi: 10.1348/000712601162293 11534740

[131] Kuhbandner C, Zehetleitner M. Dissociable effects of valence and arousal in adaptive executive control. García AV, editor. PLoS One. 2011;6: e29287. doi: 10.1371/journal.pone.0029287 22216233 PMC3244450

[132] Lang PJ, Greenwald MK, Bradley MM, Hamm AO. Looking at pictures: affective, facial, visceral, and behavioral reactions. 1993. 10.1111/j.1469-8986.1993.tb03352.x 8497555

[133] Pratto F. Consciousness and Automatic Evaluation. The Heart’s Eye. Elsevier; 1994. pp. 115–143. doi: 10.1016/b978-0-12-410560-7.50012–0

[134] Schimmack U. Attentional interference effects of emotional pictures: Threat, negativity, or arousal? Emotion. 2005;5: 55–66. doi: 10.1037/1528-3542.5.1.55 15755219

[135] Costanzi M, Cianfanelli B, Saraulli D, Lasaponara S, Doricchi F, Cestari V, et al. The Effect of Emotional Valence and Arousal on Visuo-Spatial Working Memory: Incidental Emotional Learning and Memory for Object-Location. Front Psychol. 2019;10: 2587. doi: 10.3389/fpsyg.2019.02587 31803120 PMC6877739

[136] Finucane AM, Whiteman MC, Power MJ. The effect of happiness and sadness on alerting, orienting, and executive attention. J Atten Disord. 2010;13: 629–639. doi: 10.1177/1087054709334514 19448148

[137] Frühholz S, Fehr T, Herrmann M. Early and late temporo-spatial effects of contextual interference during perception of facial affect. Int J Psychophysiol. 2009;74: 1–13. doi: 10.1016/j.ijpsycho.2009.05.010 19470392

[138] Citron FMM, Weekes BS, Ferstl EC. Effects of valence and arousal on written word recognition: Time course and ERP correlates. Neurosci Lett. 2013;533: 90–95. doi: 10.1016/j.neulet.2012.10.054 23142715

[139] West R. Neural correlates of cognitive control and conflict detection in the Stroop and digit-location tasks. Neuropsychologia. 2003;41: 1122–1135. doi: 10.1016/s0028-3932(02)00297-x 12667546

[140] Hagit M, Asher C, Leon D. Modulation of conflict related ERP components in the stroop and flanker tasks by nature of the response conflict. Front Hum Neurosci. 2011;5. doi: 10.3389/fnhum.2011.00005 21441977 PMC3031991

[141] Imbir K. Affective norms for 718 polish short texts (ANPST): Dataset with affective ratings for valence, arousal, dominance, origin, subjective significance and source dimensions. Front Psychol. 2016;7. doi: 10.3389/fpsyg.2016.00007 27458420 PMC4930931

[142] Imbir K. Arousal and Subjective Significance Shapes Stimuli Interpretation across Warmth Vs. Competence Dimensions. Curr Psychol. 2017. doi: 10.1007/s12144-016-9553-9 30416322 PMC6208855

[143] Imbir K, Spustek T, Duda J, Bernatowicz G, Zygierewicz J. N450 and LPC event-related potential correlates of an Emotional Stroop Task with words differing in valence and emotional origin. Front Psychol. 2017;8. doi: 10.3389/fpsyg.2017.00008 28611717 PMC5447706

[144] Imbir KK, Duda-Goławska J, Pastwa M, Jankowska M, Żygierewicz J. Event-Related Potential Correlates of Valence, Arousal, and Subjective Significance in Processing of an Emotional Stroop Task. Front Hum Neurosci. 2021;15: 68. doi: 10.3389/fnhum.2021.617861 33716692 PMC7947367

